# Multiple Interventions for Diabetic Foot Ulcer Treatment Trial (MIDFUT): study protocol for a randomised controlled trial

**DOI:** 10.1136/bmjopen-2019-035947

**Published:** 2020-04-19

**Authors:** Sarah Brown, Jane Nixon, Myka Ransom, Rachael Gilberts, Nikki Dewhirst, Elizabeth McGinnis, Roberta Longo, Frances Game, Chris Bojke, Paul Chadwick, Akila Chandrasekar, Ian Chetter, Howard Collier, Catherine Fernandez, Shervanthi Homer-Vanniasinkam, Edward Jude, Richard Leigh, Richard Lomas, Peter Vowden, James Wason, Linda Sharples, David Russell

**Affiliations:** 1 Clinical Trials Research Unit, Leeds Institute of Clinical Trials Research, University of Leeds, Leeds, UK; 2 Leeds Teaching Hospitals NHS Trust, Leeds, UK; 3 Academic Unit of Health Economics, Leeds Institute of Health Sciences, University of Leeds, Leeds, UK; 4 Derby Teaching Hospitals NHS Fundation Trust, Derby, UK; 5 College of Podiatry, London, UK; 6 NHS Blood and Transplant, Liverpool, UK; 7 University of Hull, Hull, UK; 8 Tameside General Hospital, Manchester, UK; 9 Royal Free London NHS Foundation Trust, London, UK; 10 Bradford Teaching Hospitals NHS Foundation Trust, Bradford, UK; 11 MRC Biostatistics Unit, University of Cambridge, Cambridge, UK; 12 Population Health Sciences Institute, Newcastle University, Newcastle upon Tyne, UK; 13 Department of Medical Statistics, London Schoool of Hygience and Tropical Medicine, London, UK

**Keywords:** diabetic foot, diabetes & endocrinology, wound management, statistics & research methods, clinical trials

## Abstract

**Introduction:**

Diabetes affects more than 425 million people worldwide with a lifetime risk of diabetic foot ulcer (DFU) of up to 25%. Management includes wound debridement, wound dressings, offloading, treatment of infection and ischaemia, optimising glycaemic control; use of advanced adjuvant therapies is limited by high cost and lack of robust evidence.

**Methods and analysis:**

A multicentre, seamless phase II/III, open, parallel group, multi-arm multi-stage randomised controlled trial in patients with a hard-to-heal DFU, with blinded outcome assessment. A maximum of 447 participants will be randomised (245 participants in phase II and 202 participants in phase III). The phase II primary objective will determine the efficacy of treatment strategies including hydrosurgical debridement ± decellularised dermal allograft, or the combination with negative pressure wound therapy, as an adjunct to treatment as usual (TAU), compared with TAU alone, with patients randomised in a 1:1:1:2 allocation. The outcome is achieving at least 50% reduction in index ulcer area at 4 weeks post randomisation.

The phase III primary objective will determine whether one treatment strategy, continued from phase II, reduces time to healing of the index ulcer compared with TAU alone, with participants randomised in a 1:1 allocation. Secondary objectives will compare healing status of the index ulcer, infection rate, reulceration, quality of life, cost-effectiveness and incidence of adverse events over 52 weeks post randomisation. Phase II and phase III primary endpoint analysis will be conducted using a mixed-effects logistic regression model and Cox proportional hazards regression, respectively. A within-trial economic evaluation will be undertaken; the primary economic analysis will be a cost-utility analysis presenting ICERs for each treatment strategy in rank order of effectiveness, with effects expressed as quality-adjusted life years.

The trial has predefined progression criteria for the selection of one treatment strategy into phase III based on efficacy, safety and costs at 4 weeks.

**Ethics and dissemination:**

Ethics approval has been granted by the National Research Ethics Service (NRES) Committee Yorkshire and The Humber - Bradford Leeds Research Ethics Committee; approved 26 April 2017; (REC reference: 17/YH/0055). There is planned publication of a monograph in National Institute for Health Research journals and main trial results and associated papers in high-impact peer-reviewed journals.

**Trial registration number:**

ISRCTN64926597; registered on 6 June 2017

**Strengths and limitations of this study**

- The multi-arm multi-stage design will allow early evaluation of multiple treatment strategies in a phase II/III design, stopping treatments which fail to demonstrate sufficient improvement, evaluating only those showing greatest efficacy in a phase III trial.
- Comparison of multiple treatment strategies to a shared control group, thus requiring fewer participants compared with conventional trial designs.
- Clear predefined progression criteria for the selection of the treatment strategy into phase III.
- Pragmatic in the identification of patients with hard to heal ulcers.
- The target sample size allows only one treatment strategy to be taken forward into phase III.

## Introduction

Diabetes currently affects more than 425 million people worldwide,^1^ and this is expected to increase to 629 million by 2045.^2^ A total of 21%–30% of patients with diabetes develop peripheral neuropathy or lose sensation in their feet^3 4^ and extrapolation from incidence studies suggests that lifetime incidence of diabetic foot ulcer (DFU) may be as high as 25%.^5^ More than 50% of DFUs become infected, requiring hospitalisation, and 20% of infections result in amputation,^6^ contributing approximately 80% of non-traumatic amputations performed in the developed world.^7^

In the UK, diabetes affects 4.5 million people^8^ with approximately 64 000 having a DFU at any one time.^9^ In 2014–2015 National Health Service (NHS) England spent an estimated £1 billion on DFU treatment.^10^ This does not take into account the costs imposed on the public sector and society as a whole through working days lost, reductions in tax revenue, increases in benefit payments and social care resources. Furthermore, DFUs have a major impact on patient health-related quality of life (HRQoL), including impaired physical function, mental well-being and social interaction.^11^

Management of DFUs comprises provision of National Institute for Health and Care Excellence (NICE) recommended best ‘treatment as usual’ (TAU) through multidisciplinary team (MDT) DFU clinics (podiatrists, diabetologists, vascular surgeons etc) and concomitant treatment strategies including: optimising glycaemic control, sharp non-surgical debridement, dressing application, off-loading, treatment of infection and ischaemia.^12^ There are a number of advanced/adjuvant therapies but their use is limited by high unit cost and an absence of robust evidence.

Despite implementation of MDT care, a national audit of over 33 000 ulcers reports 48.3% remain unhealed at 12 weeks.^13^ Of those patients with less than the median reduction in DFU area at 4 weeks (<53% reduction), only 9% went onto heal at 12 weeks,^14^ while those in the top half for healing at 4 weeks (≥53% reduction) had a higher probability of healing of 58% at 12 weeks. Delayed healing increases the probability of adverse sequelae including infection and amputation.^6^ Drivers of the cost of care change with the need for hospitalisation: in a recent study community nurse visits accounted for 65% of total costs for healed and unhealed wounds managed in the outpatient setting, whereas 65% of costs in patients having amputation were incurred in secondary care.^15^ Thus implementation of adjuvant therapies, which are often more costly, is more likely to be cost-effective in those patients identified as ‘hard to heal’ (failing to decrease by >50% at 4 weeks), whereas those DFUs reaching >50% healing at 4 weeks are likely to heal without the need for more expensive interventions. Those DFUs reported as unhealed at 12 weeks in the 2019 UK National Diabetic Foot Audit^13^ may have benefited from such therapies.

Establishing efficacious adjuvant therapies for use in non-healing wounds is a priority for improving healing rates and HRQoL, and reducing the risk of morbidity and cost. There is a paucity of high-quality trials assessing adjuvant wound therapies in DFUs, and NICE guideline NG19 and others have highlighted the need for randomised controlled trials (RCTs) of negative pressure wound therapy (NPWT) and other adjuvant therapies.^12 16^ Technological advances in three adjuvant therapies mean they are now available for routine clinic use.

1. NPWT is available in a small portable pump which doesn’t restrict patient movement.
2. Sufficient surgical debridement which changes a chronic wound biology to an acute wound can now be undertaken using hydrosurgical debridement (HD) under local anaesthetic in clinic, enhancing patient experience and reducing costs by avoiding additional hospital visits for day-case surgery. This also allows wound bed preparation to a ‘graft ready state’ for advanced wound adjuncts, a state which cannot be achieved by less aggressive debridement with wound debridement pads such as Debrisoft. HD has been shown to be as effective as operating theatre surgical debridement in wound healing outcomes.^17^
3. Decellularised dermal allograft (DCD) has been used in the USA for treatment of DFU, with improved healing compared with standard care^18 19^ but cost has been prohibitive in the UK. A novel DCD, prepared from skin donated by voluntary UK deceased donors and recently been developed within the NHS, is approved by the Human Tissue Authority and is available for use in the UK. DCD is prepared and supplied by NHS Blood and Transplant (a Department of Health special health authority). However, the application of DCD requires surgical debridement to a ‘graft ready wound bed’ and it is not known whether the surgical debridement alone leads to improved healing in this setting.

Performing multiple RCTs to assess each intervention individually would be time-consuming and expensive. Moreover, these therapies are often used in combination. The Multiple Interventions for Diabetic Foot Ulcer Treatment Trial (MIDFUT) uses an efficient, informative and ethical, adaptive, multi-arm, multi-stage (MAMS) design.^20^ This involves early evaluation of combinations of the candidate interventions in a phase II/III design, stopping recruitment to treatment strategies which fail to demonstrate sufficient improvements in DFU healing, using an intermediate endpoint at 4 weeks post randomisation, and evaluating only one treatment strategy showing greatest efficacy in a phase III trial.

The evidence for adjuvant therapies for DFU treatment was reviewed in NICE guideline NG19,^12^ which concluded that the quality of trials was poor due to binary and early endpoints and small sample sizes. It recommended that future trials are sufficiently powered, with outcomes including time to healing, incidence and extent of amputation (major or minor), ulcer recurrence, HRQoL, adverse events (AEs), hospital admissions and length of stay. These findings were supported by a review by the International Working Group on the Diabetic Foot.^21^

In summary, quality data on the outcome of adjuvant therapies for DFU is rare. A 30% increase in prevalence of disease is anticipated by 2035, which will add to the already substantial costs of treating DFUs, in a time of global fiscal uncertainty. With international guidance advocating the need for robust RCTs in this area,^21^ and technical advancements in three adjuvant therapy options (HD, DCD and NPWT), an RCT to compare treatment strategies is timely.

### Objectives

In phase II, the aim is to identify the most promising of the three treatment strategies compared with TAU using short-term efficacy and in phase III, to investigate the clinical and cost effectiveness of one treatment strategy from phase II compared with TAU in the treatment of patients with hard-to-heal DFUs.

#### Phase II primary objective

To determine the efficacy of the treatment strategies:

1. TAU + HD alone.
2. TAU +HD + DCD.
3. TAU +HD + NPWT+ DCD

compared with TAU alone using the short-term intermediate outcome of achieving at least 50% reduction in index ulcer area at 4 weeks post randomisation (phase II primary endpoint).

#### Phase III primary objective

To determine whether one treatment strategy continued from phase II, as an adjunct to TAU, reduces time to healing of the index ulcer compared with TAU alone.

The primary endpoint is time *to healing of the index ulcer* from randomisation to the date the index ulcer is confirmed as healed at the first confirmation visit conducted by the blinded assessor (providing the index ulcer is confirmed as healed at a second clinical assessment 2 weeks later).

#### Phase III secondary objectives

To compare one treatment strategy as an adjunct to TAU, continued from phase II, with TAU alone for:

- Healing status of the index ulcer at 12, 20 and 52 weeks.
- Rate of ulcer infection in the foot of the index ulcer over 52 weeks post randomisation.
- Incidence of reulceration following healing of index ulcer over 52 weeks post randomisation.
- Quality of life using the Diabetic Foot Ulcer Scale—Short Form (DFS-SF) and the Five-level five-dimension EuroQol (EQ-5D-5L) over 52 weeks post randomisation.
- Incidence of AEs (including amputation, infection in any ulcer on the foot of the index ulcer and hospital admission) over 52 weeks post randomisation.
- Cost effectiveness over 52 weeks.

#### Phase III exploratory objective

To explore factors prognostic of ulcer healing.

## Methods

MIDFUT is a multicentre, seamless phase II/III, open, parallel group, MAMS RCT in patients with a hard-to-heal DFU, with blinded outcome assessment.

**Figure 1:**
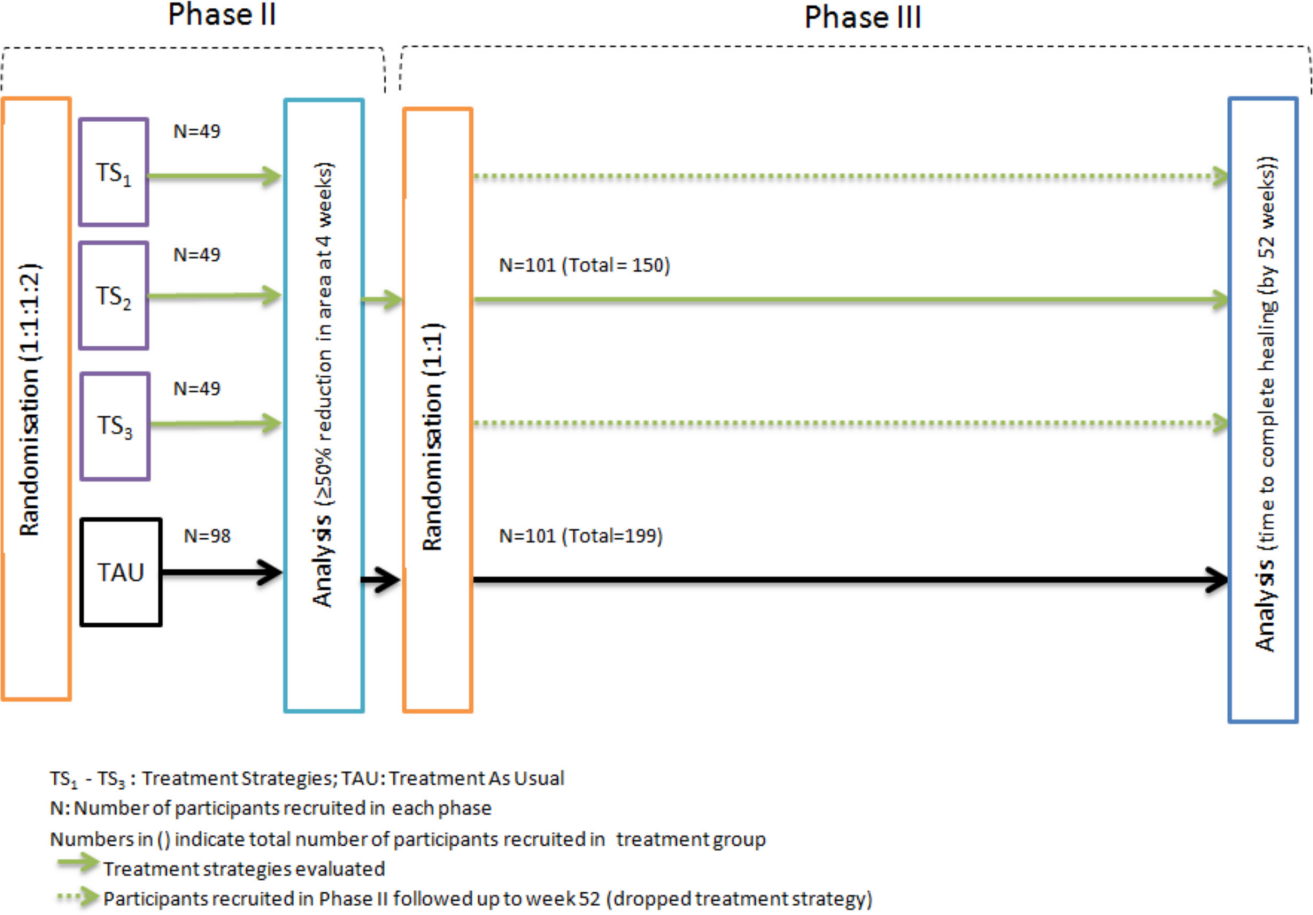
Multiple Interventions for Diabetic Foot Ulcer Treatment Trial (MIDFUT) design.

The MAMS trial design will allow an early evaluation of three candidate treatment strategies in a phase II/III design. Randomisation to treatment strategies that fail to demonstrate sufficient improvement in index ulcer healing at the end of phase II will be stopped. Only one treatment strategy showing greatest early efficacy will undergo clinical and cost-effectiveness assessment in phase III (see figure 1 for a schematic of the design).

Three treatment strategies will be compared with TAU in phase II. Treatment strategies for which the estimated proportion of responders is less than 10% higher than the proportion of responders on TAU (absolute difference) will be dropped at the end of phase II (response defined as achieving at least 50% reduction in wound area of the index ulcer). One treatment strategy and TAU will be evaluated in phase III. If more than one treatment strategy shows a sufficient response in phase II, then the decision on which treatment strategy to evaluate in phase III will consider information on the safety profile and costs of the treatment strategies up to 4 weeks post randomisation.

A maximum of 447 participants will be recruited, 245 participants in phase II and at most 202 participants in phase III. Recruitment at centres will continue without interruption between phase II and phase III.

**Figure 2:**
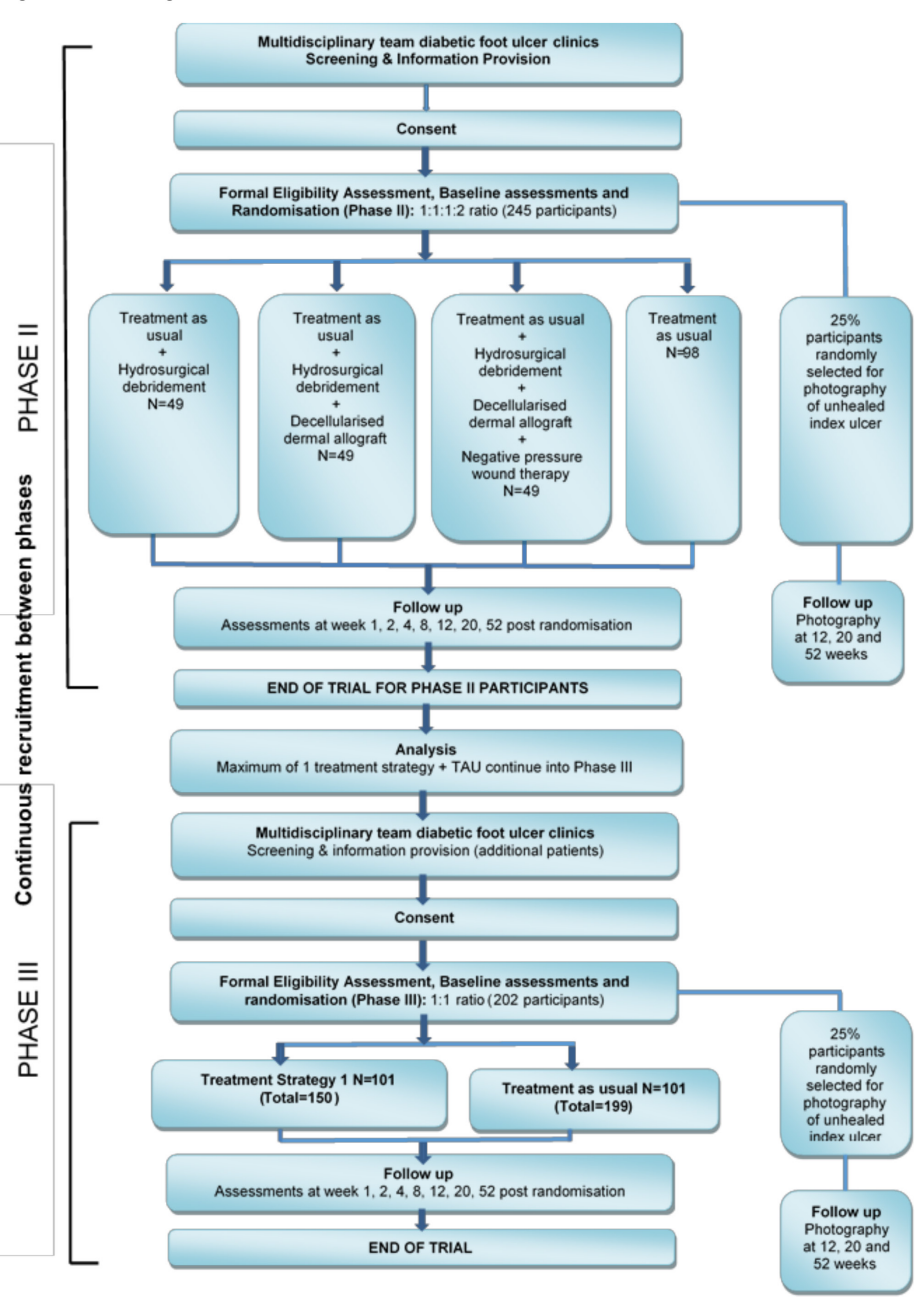
Flow diagram. TAU, treatment as usual.

All participants from phases II and III will be followed up at weeks 1, 2, 4, 8, 12, 20 and 52 post randomisation (including those where healing of the index ulcer has been confirmed), or week 54 where healing of the index ulcer is first reported at week 52 (see figure 2).

The trial includes a 9-month internal pilot phase in phase II to evaluate the feasibility of recruitment and therefore delivery of the trial.

Moreover, a Study Within A Trial will be included to determine the extent of agreement in the assessment of healing between central blinded photography review and the clinical assessment of healing.

An interim analysis will be conducted after 220 participants have reached 52 weeks post randomisation to re-estimate the overall loss to follow-up rate and the final sample size. The review will be conducted in a blinded manner.

### Recruitment/consent

The trial will be conducted in secondary care and community clinics that provide a MDT DFU service (which includes as a minimum a clinician trained in each trial intervention, a podiatrist, a diabetologist, a vascular surgeon and an orthotist).

Patients under the care of the MDT DFU outpatient clinics, with a current DFU, surgical debridement wound or minor amputation wound, will be assessed for eligibility in accordance with the criteria in box 1.

Box 1 **Inclusion and exclusion criteria**

**Inclusion criteria**

- Aged ≥18 years.
- Diagnosis of diabetes mellitus (according to WHO criteria).^47^
- Has a chronic diabetic foot ulcer (DFU) or surgical debridement wound or open minor amputation and in the opinion of the attending clinical team is not on a healing trajectory despite usual best care for a minimum of 4 weeks since initial presentation at the multidisciplinary team (MDT) DFU service.*
- The index DFU has an area ≥0.8 cm^2^.
- Ankle brachial index for the leg of the index ulcer ≥0.7 or non-compressible (measurements available in the participants notes taken within 3 months of randomisation can be used if no change in intervention or vascular events have occurred).
- Expected to comply with the treatment strategies and follow-up schedule.
- Consent to foot and wound photography.
- Consent to participate (written/witnessed verbal informed consent).

**Exclusion criteria**

- Has any current clinically infected DFU on the foot of the index ulcer (as per Infectious Diseases Society of America (IDSA) guidelines).^48^
- Glycated haemoglobin (HbA1c)>110 mmol/mol (measurements available in the participants notes taken within 3 months of randomisation can be used if no change in intervention or vascular events have occurred).
- Estimated glomerular filtration rate (eGFR) <20 mL/min/1.73 m^2^ (measurements taken within 3 months of randomisation can be used if no change in intervention or vascular events have occurred).
- Index ulcer duration >2 years.
- Planned or previous treatment with corticosteroids to an equivalent dose of prednisolone >10 mg per day or other immunosuppressive/immunomodulating therapy within 4 weeks prior to randomisation.
- Has evidence of connective tissue disorders as a cause of ulceration (eg, vasculitis or rheumatoid arthritis).
- Has evidence of dermatological disorders as a cause of ulceration (eg, pyoderma gangrenosum or epidermolysis bullosa).
- Planned or previous growth factor treatment within 4 weeks prior to randomisation.
- Planned or previous revascularisation or foot surgery affecting healing on the foot of the index ulcer within the 4 weeks prior to randomisation.
- Index ulcer base has bone or joint involvement.
- Previously received decellularised dermal allograft (DCD) for the index ulcer within 4 weeks prior to randomisation.
- Previously received negative pressure wound therapy (NPWT) for the index ulcer within 4 weeks prior to randomisation.
- Previously received hydrosurgical or surgical debridement for the index ulcer within 4 weeks prior to randomisation.
- Has previously been randomised to the Multiple Interventions for Diabetic Foot Ulcer Treatment Trial (MIDFUT) Study.
- Unable to receive one or more of the randomised treatment strategies for any reason at the discretion of the attending clinical team (eg, risk of excessive bleeding, serious falls risk, known allergies to NPWT dressings or dCELL dermis preparation components).

*Defined as failure to achieve >50% reduction in index ulcer area over a minimum of 4 weeks using local wound measurement policies.

Potentially eligible patients will receive a verbal explanation of the study and a patient information leaflet (PIL) by the attending clinical/research team. Strategies to encourage recruitment include:

- Posters and/or leaflets in clinic waiting areas and other appropriate locations.
- Letter and PIL sent to patients with their outpatient appointment letter.
- Study included on relevant websites and research databases that can be accessed by members of the public.
- Ethically approved tweets on Twitter.

Following information provision, patients will have as long as they need to consider participation and to discuss the study with their family and other healthcare professionals before consent to participate in the study is requested.

Assenting patients will be invited to provide informed consent and complete an eligibility assessment. Full informed consent will be obtained for all participants prior to the participant undergoing procedures that are specifically for the purposes of the study and are not part of TAU at the participating centre.

Witnessed consent by a representative who is independent of the trial will be available where relevant.

Patients who provide written/witnessed verbal informed consent, but subsequently lose capacity will be withdrawn from the trial.

### Non-randomised patients

Participating research sites will complete a log of all patients presenting with a DFU and considered for the trial, but not recruited. Anonymised information to be collected includes age, sex, ethnicity, reason not eligible or reason declined participation.

### Randomisation

Following confirmation of eligibility, consent and completion of baseline assessments, participants will be randomised. In phase II, randomisation will be in a 1:1:1:2 allocation ratio to the three treatment strategies and TAU group, respectively, as an approximation to Dunnett’s recommendation.^22^ In phase III, randomisation will be in a 1:1 allocation ratio to one treatment strategy and TAU. Randomisation in both phases will use a minimisation algorithm, incorporating a random element, via a central 24 hours automated telephone or internet randomisation system, based at the Leeds Clinical Trials Research Unit (CTRU). The dynamic allocation method will ensure the groups are well balanced for:

- Centre.
- Aetiology (neuropathic or neuro-ischaemic).
- Index ulcer duration (<6 months, ≥6 months).
- Anatomical site (forefoot, midfoot/hindfoot).
- Presentation (DFU, surgical debridement wound, open minor amputation).

In addition, at the time of randomisation, 25% of participants will be randomly selected to have photographs of the index ulcer taken, if it is unhealed, at weeks 12, 20 and 52, for central blinded review.

### Blinding

Due to the nature of the treatment strategies it is not possible to blind participants, the clinicians or research team to the treatment group allocation. However, primary outcome assessments will be conducted by an independent clinical assessor with no knowledge of treatment allocation. To mitigate risk of assessment bias the blinded assessor will also have no access to participant notes or trial case report forms. Blinding will be maintained when tracings and photographs at week 4, and confirmation of the index ulcer healing assessments, are returned to the CTRU (eg, through separate mail or independent clerical staff).

For the phase II primary outcome and phase III exploratory objective, a blinded assessor at each site will complete an acetate tracing and take a two-dimensional digital photograph of the index ulcer at week 4. Measurements will be obtained from the index ulcer tracing using ‘Image J’ software^23^ by a member of the CTRU team who is independent of the research teams at recruiting sites and blind to treatment allocation. A photograph of the index ulcer will be taken as a backup in the event that a tracing cannot be taken or is of insufficient quality to determine the index ulcer outline.

For the phase III primary endpoint, all participants recruited to both phase II and III will also have a photograph taken of the reported healed index ulcer by the blinded assessor within 3 days of healing being reported and 2 weeks later as a confirmation of healing, which will undergo blinded central review.

Photographs taken of healed and of unhealed index ulcers for randomly selected participants will be submitted for central blinded photography review by clinical members of the Trial Management Group who will not be aware of the participant’s identity, treatment group or time point at which the photograph was taken.

### Interventions

All randomised treatment strategies will be applied to the index ulcer as a ‘once only intervention’ on the day of randomisation in the MDT DFU service clinic, with the exception of NPWT which will be applied until the 2 week visit. Treatment of any other ulcers will continue as per the treating clinician’s decision.

At baseline, randomisation and each follow-up visit all participants will receive TAU. At the randomisation treatment visit, the participant will be randomised to receive the treatment strategy specific to the arm of the trial for the index ulcer. This will include one or more of the following:

#### Treatment as usual

Participants will receive the minimum standard care provided by the recruiting centre. This will be in line with NICE guidelines^12^ and is likely to include attendance at the MDT DFU service clinic(s) at least fortnightly until healing is confirmed for wound assessment, sharp non-surgical debridement of callous/non-viable tissue, review of off-loading and to optimise diabetes and wound assessment as required, including community services visits, typically once to two times weekly. In line with NICE guidelines, use of removable below-knee walking device or removable cast walker will be encouraged. Wound dressing changes will be performed between clinic visits according to local policies.

#### Hydrosurgical debridement

HD, a one-off procedure on the day of randomisation, applies saline at high pressure via a pump through a hand piece. This has an operating window located at the instrument’s distal tip. During operation the flow of pressurised saline creates a local vacuum. As the operating window of the handset is passed over the tissue, non-viable material and debris are removed. The ulcer bed is debrided to healthy bleeding tissue which may require local anaesthetic.

#### Negative pressure wound therapy

NPWT, applied on the day of randomisation, consists of a foam dressing cut to shape and applied to the wound. An airtight seal is established with a film dressing; this is then connected to a pump which applies gentle suction to the wound. This allows the removal of fluid from the wound, which is collected in a canister attached to the pump, which is carried by the participant at all times in the bag provided. Alternatively, a self-contained system consisting of a disposable NPWT pump attached to an absorbent adhesive dressing may be used. The dressing is usually changed at least once a week, and the NPWT will be applied for 2 weeks post randomisation.

#### Decellularised dermal allograft

DCDs are prepared from split skin grafts obtained from deceased human tissue donors, which are processed and sterilised. Processing retains the normal skin structure, but removes donor cells and cell remnants meaning the graft is not rejected and functions as a permanent tissue replacement. On receipt at participating sites, the graft will be stored between 0°C and 40°C until the expiry date stated on the graft label. Prior to application the graft is soaked in a bowl of sterile saline solution for 15 min. The graft is then cut to size using sterile scissors and applied directly to the debrided wound bed, epidermal side upwards. Following application, the ulcer is covered with a non-adherent contact layer and a secondary bolster dressing or NPWT (as per randomisation). In those DFUs allocated to DCD, the wound bed is not debrided for 4 months post-treatment, unless clinically indicated, although debridement of wound edge and surrounding tissue can continue as per TAU.

### Assessments/data collection and follow-up

#### Baseline assessment

Participant demographics including date of birth, gender, ethnicity, NHS number and site of the index ulcer will be recorded.

Clinical history will be recorded including smoking history, duration and type of diabetes, number of ulcers and index ulcer characteristics, for example, first or recurrent ulcer, aetiology, existing wound therapies and Site, Ischaemia, Neuropathy, Bacterial Infection and Depth (SINBAD) classification.^24^ Initial index ulcer area tracing (using acetate) will also be obtained. Participants will be asked to complete quality of life questionnaires: DFS-SF and EQ-5D-5L.

Randomisation and application of the treatment strategy will take place after baseline assessments and questionnaires have been completed, on the same day.

Information collected post-treatment will include details of the treatment strategy applied to the index ulcer and to any other ulcers on the foot of the index ulcer, and expected AEs and serious AEs (SAEs). Post HD debridement, index ulcer area acetate tracing and photographs will be obtained.

#### Follow-up assessments

At a routine clinic assessment at week 1 and at weeks 2, 4, 12, 20 and 52 post randomisation the following assessments will be conducted by a member of the clinical research team (clinician, clinical research nurse or registered healthcare professional): healing status of the index ulcer, episodes of infection in the foot of the index ulcer (IDSA criteria), revascularisation of the index limb, index ulcer treatments and expected AEs or SAEs. In addition, at weeks 1 and 2 post randomisation, an assessment of compliance with NPWT and DCD (where applicable), and at weeks 2–52 post randomisation an assessment of reulceration of the index ulcer will be conducted. At week 2 and week 4 post randomisation, an acetate tracing and photograph of the index ulcer post sharp non-surgical debridement (where clinically indicated) will be taken; at week 4 this is conducted by a blinded assessor (clinician, research nurse or registered healthcare professional).

Participant questionnaires (DFS-SF, EQ-5D-5L and healthcare resource utilisation (HRU)) will be completed at weeks 4, 12, 20 and 52 post randomisation; the HRU questionnaire will also be completed at week 8 post randomisation.

#### Healing and reulceration assessments

Healing is defined as complete closure of the ulcer: 100% re-epithelialisation of the wound surface with the absence of drainage, confirmed by blinded assessment of index ulcer healing status at two consecutive assessments 2 weeks apart.^25^

Healing of the index ulcer will be reported in one of the following scenarios:

- By the research nurse/registered healthcare professional at a research visit.
- During the participant’s routine appointment at the MDT DFU service clinic, podiatry clinic, GP practice nurse and/or at home by district nurses as per TAU.
- Participant self-reporting to the research team or to the attending clinical team in between routine appointments who will then inform the research team.

The attending clinical team will contact the research team to report the date the index ulcer was first noted as healed, who will then arrange an initial visit within 3 days of healing of the index ulcer first being reported and a 2-week follow-up visit (±3 days) with the blinded assessor to assess index ulcer healing status and conduct photography.

Reulceration is defined as recurrence of a full thickness break in the epithelium at the same location as the index ulcer.^26^ Reulceration of the index ulcer will be established either by participant self-referral to the research team, at a routine clinic or research appointment or by continuous screening of new referrals to the MDT DFU service clinic where participants will be flagged to the research team by the attending clinical team. Reulceration of the index ulcer will be confirmed by a blinded assessor, within 7 days of reulceration being reported, with reference to the photograph of the foot taken at the randomisation visit, photography undertaken and the date of reulceration of the index ulcer recorded.

### Sample size

The planned maximum sample size is 447 participants, 245 participants in phase II and 202 participants in phase III. The apportionment of participants to phase II and phase III was estimated using a series of simulation studies.

In phase II, 49 participants per treatment strategy arm and 98 participants in the TAU arm will be recruited. The target effect size in phase II is an absolute increase of 25% in the proportion of participants achieving at least a 50% reduction in wound area by 4 weeks post randomisation, assuming 39% reach at least a 50% reduction by week 4 in the TAU arm (local audit data) and 64% achieve this outcome in the treatment strategy arms.

An additional 101 participants will be recruited into each arm evaluated in phase III, corresponding to a total (phase II and III combined) of 150 in the remaining treatment strategy group and 199 in the TAU arm (total of 349 participants for evaluation in phase III).

The minimum clinically important effect size in phase III is a hazard ratio (HR) of 1.5, assuming a median time to healing of 21 weeks for the TAU arm (local audit data) and 14 weeks for the treatment strategy arms^18 27–30^ and 18.0% and 7.6% *un*healed at 52 weeks in the TAU and treatment strategy groups, respectively (assuming exponential distribution for time to healing).

Several scenarios for the power of the trial have been considered. In all cases a 10% loss to follow-up by 4 weeks and 25% loss to follow-up by 52 weeks is assumed. In the case where there is a single effective treatment strategy arm, the design has 83.9% power to recommend a truly effective treatment strategy (ie, for it to progress from phase II and for a significant result found at phase III). A treatment strategy group that progresses to phase III and which is significantly better than TAU at the two-sided 4% significance level (to control the familywise error rate at 5%) on the time to healing endpoint will be declared clinically effective.

A formal sample size review will be conducted at 52 weeks, after 220 participants have been recruited, to re-estimate the proportion of participants lost to follow-up by 52 weeks post randomisation and the final sample size. The review will allow the overall loss to follow-up to be estimated to a minimum precision ±5.7% (corresponding to half width of the 95% CI), assuming a maximum loss to follow-up of 25%.

### Progression criteria for phase III

The minimum criterion for taking treatment strategies forward into phase III will be defined as at least a 10% increase in the probability of achieving ≥50% reduction in index ulcer area at 4 weeks post randomisation above that observed for TAU, corresponding to the minimum clinically important difference (clinical opinion). If more than one treatment strategy passes this threshold at phase II then the selection criteria will be based on a combination of efficacy, safety profile and cost of treatment strategies up to 4 weeks post randomisation. The progression criteria are provided in further detail in box 2.

Box 2 **Phase II to phase III progression criteria**

The progression criteria for the selection of treatment strategies into phase III is based on the following process:

- Calculate the point estimate for the proportion of participants with ≥50% ulcer area reduction at 4 weeks post randomisation in all four arms (phase II endpoint). Drop treatment strategy arms for which this proportion is less than 10% higher (on the absolute scale) than that of TAU. That is, if the proportion for TAU is 39% then recommend dropping treatment strategies for which the proportion is less than 49%. Rank the remaining treatment strategies in order of clinical activity.

Note: An absolute improvement of 10% in the proportion of participants with ≥50% ulcer area reduction at 4 weeks post randomisation corresponds to the critical cut point for the selection of treatment strategies based on clinical activity.

- If more than one treatment strategy has a success rate at 4 weeks of at least 10% greater than TAU then summarise SAEs and rank order of treatment strategies in terms of their safety profile. Only AEs and SAEs that are classified as expected and related to DFUs or trial treatment strategies, or ‘related and unexpected SAEs’ (RUSAEs) will be considered. Decision on whether to drop treatment strategies with the ‘least favourable’ safety profile will be made by the Data Monitoring and Ethics Committee (DMEC).
- If more than one treatment strategy remains after stages (1) and (2) then summarise treatment-related costs up to 4 weeks and rank order of treatment strategies by ascending cost of treatment versus TAU. Decision on whether to drop the treatment strategies with the highest cost will be made by the DMEC.
- If no treatment strategies remain, then recommend terminating recruitment to the trial. If one remains, take this forward to phase III. If more than one treatment strategy satisfies (1) to (3), take forward the top performing arm defined by clinical activity (the extent of improvement in the proportion of participants achieving the phase II endpoint).

The trial will also have a futility rule to allow for stopping of the trial on the basis of no treatment strategy demonstrating at least 10% absolute improvement in the success rate of the phase II primary outcome. This will be non-binding to allow the DMEC to make the final recommendations to the Trial Steering Committee (TSC) on whether or not to stop the trial.

### Statistical analysis

A statistical analysis plan for phase II and phase III final analyses will be finalised and signed off before any data analyses are conducted.

The complete case population will be used for the analysis of the phase II endpoint under the assumption that data are missing at random (MAR).^31^ A sensitivity analysis will be considered if there is differential missing endpoint data observed across treatment arms.

Phase III analyses will use intention to treat whereby participants will be analysed according to randomised treatment group. A per-protocol population will also be defined.

For phase III endpoint analyses, data from all participants recruited in phase II and phase III will be included.

#### Phase II primary endpoint analysis

##### Primary analysis

Treatment effects and 95% confidence intervals on response at 4 weeks will be estimated from multivariable mixed-effects logistic regression, including the minimisation factors and treatment group as fixed effects and centres as random effects. Simple contrasts for each treatment strategy compared with the TAU arm will be used.

#### Phase II secondary endpoint analysis

AEs and SAEs that are classified as expected and related to DFUs and trial treatment strategies, or RUSAEs will be summarised by treatment group.

Mean per-patient costs of treatment, healthcare use and total resource use, together with a measure of variance will be reported by treatment group.

#### Phase III primary endpoint analysis

##### Primary analysis

The hazard ratios for the phase III endpoint will be estimated using Cox proportional hazards regression with covariates for the minimisation factors, treatment arm (fixed effects) and centre (random effects) and stratification for the phase in which the participant was recruited.

#### Phase III secondary endpoint analysis

Similar regression-based analyses will be used for other secondary endpoints. Cumulative incidence of healing at 12, 20 and 52 weeks post randomisation will be obtained from the primary endpoint analysis model. A Poisson-Gamma regression model will be fitted to infection status over time. A Cox proportional hazards regression model will be fitted to time to reulceration of the index ulcer on those participants where healing of the index ulcer is confirmed. A repeated measures, random coefficients, linear regression model will be fitted to the DFS-SF Score over time.

All AEs and SAEs, including amputations and admissions to hospital, will be recorded and summarised by treatment strategy received. Expected treatment-related AEs include: pain, bleeding and infection from HD; bleeding, infection and skin irritation/breakdown from NPWT; seroma and allergic reaction from DCD.

#### Exploratory analyses

##### Sensitivity analyses

For all analyses using the Cox proportional hazards model, the assumption of independence of the distribution of time to healing/recurrence and time to other events, that is, amputation and death will be assessed and alternative models considered if there are sufficient competing risks.

A multivariable Cox proportional hazards regression model will be fitted to explore risk factors predictive of time to healing.

### Economic evaluation

A within-trial economic evaluation will be undertaken at week 52 post randomisation. The proposed secondary endpoints and methods for the economic evaluation follow the reference case set out by NICE.^32^ The primary economic analysis will be a cost-utility analysis presenting incremental cost-effectiveness ratios (ICERs) for each treatment strategy in rank order of effectiveness, with effects expressed in terms of quality-adjusted life years (QALYs). An NHS and Personal Social Services perspective for costs will be adopted. Costs and effects for each treatment strategy will be calculated for the trial follow-up period of 52 weeks.

Health resource use questionnaires will collect information on NHS and personal social care use in line with NICE guidelines.^32^ This will include primary, secondary and community resource use. Unit cost data will be obtained from national databases such as the *British National Formulary* and *Personal Social Services Research Unit Costs of Health and Social Care*.

Treatment costs include the cost of delivering each strategy (mainly given by person-time of healthcare professionals) and the cost of the necessary equipment. The scope of resources considered includes the direct healthcare costs incurred for necessary patient care and excludes resources driven by the study protocol (eg, routine clinics will be included, while research visits that are just for checking for reulceration are excluded; also, the cost of photography and visit time for collecting data for study purposes will be excluded). To cost the treatment strategies, data on average duration of appointments for delivering the treatment strategy will be collected.

ICERs, incremental net monetary benefit and incremental net health benefit statistics will be computed. NICE considers a cost per QALY within the range of £20 000–£30 000 to be acceptable.^32^

Multiple imputation will be used to address any issues of missing data in the base case analysis on the assumption of MAR. Complete case analysis will be conducted as a sensitivity analysis.

Probabilistic sensitivity analysis will be used to assess the impact of sampling uncertainty on the within-trial evaluation results. Simulated cost and QALY estimates will be plotted on the cost-effectiveness plane to illustrate the uncertainty surrounding cost-effectiveness estimates^33^ and presented as cost-effectiveness acceptability frontier to capture the varying probability of interventions being the most cost-effective over a range of willingness to pay for QALY thresholds.^34^

In addition, alternative scenarios will be explored in the sensitivity analysis to test the robustness of the main trial analysis results.

### Data management

Data will be monitored for quality and completeness and missing data will be chased up. Data received, including photographs will be stored in a secure database at Leeds CTRU in accordance with the 2018 Data Protection Act and the General Data Protection Regulation.

### Patient and public involvement

The trial was supported at the stage of developing the grant application by the Sheffield Teaching Hospitals Lay Advisory Panel for Diabetes & Endocrinology Research. Their input was central to study design, actively helping to shape discussions and decisions through written feedback and group discussion. In particular they informed decisions on how frequently patients should be reassessed at the study site; whether patients would be willing to report healing or other outcomes directly to the research team or through their community clinician; agreement to completion of questionnaires; wound tracing and photographs which may be considered a burden; how willing patients will be to take part in the study; the acceptability of each intervention.

The trial has two Patient and Public Involvement (PPI) representatives on the Trial Steering Committee who have provided input into the patient information sheet and other trial documentation intended for use by patients. The PPI representatives also provide input into the design and conduct of the trial at 6 monthly meetings. This high-level involvement in project management aims to ensure patients’ perspectives are fully integrated in key decisions about the trial, delivery and interpretation/dissemination of findings.

## Discussion

### MAMS design

The chosen MAMS design provides an efficient platform for assessment of several competing interventions, quickly homing in on the treatment strategy with greatest potential to be effective, early dropping of ineffective treatments and allowing assessment of combinations of treatments.^20 35–37^ Specific advantages of the MAMS design include comparing multiple treatment arms to a shared control group thereby requiring fewer patients, improved consent/recruitment rates since patients are more likely to receive an active treatment^37^ and common eligibility criteria across trial arms.^20^ Moreover, recruitment to all treatment strategies will continue during analysis and reporting of phase II data to ensure no loss of momentum in recruitment at sites.

### Choice of endpoints

Reduction in ulcer area at 4 weeks was chosen as the primary outcome at phase II to be consistent with published observational studies^38 39^ and DFU RCTs.^40^ This intermediate outcome measure provides a means of screening for ‘emerging evidence’ of efficacy, as it occurs earlier and more frequently than the definitive outcome measure and that it is on the causal pathway.^41^ Thus, it allows the phase II analysis and decision on treatment selection to take place in a timely manner.

Time to healing, the primary endpoint in phase III, was chosen as an important outcome measure from both clinical and economic perspectives.^30^

Ulcer infection is the most common complication in non-healing ulcers, occurring in more than 50% of DFUs.^6^ It results in delayed healing, prolonged treatment, increased resource use and increases the risk of a patient requiring a major amputation.^42^ IDSA criteria is recognised as a gold standard for characterisation of infection in DFUs in many national and international guidelines and will provide a reproducible system for clinical diagnosis and severity stratification.^12 21 43^

No single patient-reported outcome measure has been identified as a ‘gold standard’ for assessing HRQoL in diabetes-related foot disease.^11^ As a result, both the disease-specific questionnaire, DFS-SF^44^ and the preference-based utility measure, EQ-5D-5L (www.euroqol.org),^45^ will be completed by patients. The DFS-SF questionnaire has acceptable psychometric properties for measuring quality of life for patients with DFUs. The EQ-5D is a generic instrument and forms part of the NICE reference case for cost per QALY analysis.

### Blinded assessment of healing

Having a blinded assessment of healing is important in reducing the risk of assessment bias and the trial includes independent, blinded clinical assessment of healing at both the first and confirmation of healing assessments, along with additional blinded review of photography undertaken by clinicians (Chief Investigator (vascular surgeon) and clinical nurse specialists).

### Revision to trial design

Since opening, the trial has undergone a trial redesign. The original design included a fourth treatment strategy in phase II corresponding to a combination of HD and NPWT as an adjunct to TAU, and also allowed a maximum of two treatment strategies to go forward into phase III under the same progression criteria. This original design required a maximum sample size of 660 participants, 324 participants recruited in phase II and 336 in phase III, under the sample size assumptions. Following a review of treatment strategies which would be considered in clinical practice if shown to be clinically effective and cost effective, a revised trial design dropped the combination of the HD and NPWT arm, which reduced the maximum sample size to 447 patients while still ensuring a trial of clinical relevance.

### Hard-to-heal ulcers

A registration phase was also included in the original trial design. It is important that the trial includes a patient population with ‘hard-to-heal’ ulcers, reflecting the target population that would be considered for such adjuvant therapies in clinical practice. As there is variability in usual wound area assessment and documentation across recruiting centres, the registration phase included in the original design allowed a consistent approach to assessment of healing over a 4-week run-in period, and thereby considered ‘best practice’ for trials of DFU healing. However, the recently published LeucoPatch Trial,^46^ using an identical registration phase criterion of 50% healing at 4 weeks, reported only 22% healing in the control and 34% healing in the intervention arms, suggesting that this registration phase criteria is overly selective in the definition of ‘hard-to-heal’. Early audit of data from a single centre in MIDFUT showed that patients were not recruited to the trial at presentation to an MDT DFU clinic, and had been subjected to an ‘in house assessment period’ prior to registration in the trial. Seventy-five per cent of those failing the trial registration period due to ulcer healing of >50% remained unhealed at 12 weeks, suggesting the trial was excluding a group of ulcers that were in fact ‘hard-to-heal’. The 2018 UK National Diabetic Foot Care Audit data of 21 000 ulcer episodes reports 49.3% remain unhealed at 12 weeks, with 27.3% unhealed 24 weeks and a further 2.9% recurring. Thus under half of ulcers unhealed at 12 weeks will heal in the subsequent 12 weeks, supporting a second inclusion criterion of ulcers that remain unhealed at a 12-week time point.^9^ A parallel entry route was therefore introduced to allow patients with ulcers of ≥12 weeks duration, that are considered hard to heal by the treating MDT team, to proceed directly to randomisation.

Following challenges in recruiting patients and the supporting evidence that a large proportion of patients with hard-to-heal ulcers were being missed, a decision was made to drop the registration phase for all patients. Instead, included ulcers will have failed to reduce in area by >50% over at least 4 weeks as measured using local measurement techniques. This allows a more pragmatic approach to be taken in identifying patients with hard-to-heal ulcers by using local wound measurement policies, thereby minimising the risk of missing potentially eligible patients while ensuring the trial results are more generalisable to the target patient population.

### Adjuvant therapies

It is not anticipated that there will be a rapid change in the technologies investigated in this trial, other than design changes aimed at increasing clinician and patient acceptance, again increasing the potential adoption and generalisability of the trial outcomes.

### Trial status

The first participant was registered on 10 August 2017 and the first participant randomised on 30 October 2017. As of 4 December 2019, 167 participants have been registered and 88 randomised. Recruitment is expected to complete by 31 August 2022. The full trial protocol is available on the National Institute for Health Research journals library https://www.journalslibrary.nihr.ac.uk/programmes/hta/150877/%23/.

## Supplementary Material

Reviewer comments

Author's manuscript
