# Supplementary material for: Multiple Interventions for Diabetic Foot Ulcer Treatment Trial (MIDFUT): study protocol for a randomised controlled trial
Source: BMJ Open. 2020 Apr 19;10(4):e035947. doi: 10.1136/bmjopen-2019-035947 (PMC7245399; doi:10.1136/bmjopen-2019-035947)
Supplement: Author's manuscript [file bmjopen-2019-035947.draft_revisions.pdf]

BMJ Open is committed to open peer review. As part of this commitment we make the peer review history of every article we publish publicly available.

When an article is published we post the peer reviewers' comments and the authors' responses online. We also post the versions of the paper that were used during peer review. These are the versions that the peer review comments apply to.

The versions of the paper that follow are the versions that were submitted during the peer review process. They are not the versions of record or the final published versions. They should not be cited or distributed as the published version of this manuscript.

BMJ Open is an open access journal and the full, final, typeset and author-corrected version of record of the manuscript is available on our site with no access controls, subscription charges or pay-per-view fees (http://bmjopen.bmj.com).

If you have any questions on BMJ Open's open peer review process please email info.bmjopen@bmj.com

BMJ Open

Multiple Interventions for Diabetic Foot Ulcer Treatment Trial (MIDFUT): Study Protocol for a randomised controlled trial

Journal:	*BMJ Open*
Manuscript ID	bmjopen-2019-035947
Article Type:	Protocol
Date Submitted by the Author:	25-Nov-2019
Complete List of Authors:	Brown, Sarah; University of Leeds, Clinical Trials Research Unit<br>Nixon, Jane; University of Leeds, Clinical Trials Research Unit<br>Ransom, Myka; University of Leeds, Clinical Trials Research Unit<br>Gilberts, Rachael; University of Leeds, Clinical Trials Research Unit<br>Dewhirst, Nikki; Leeds Teaching Hospitals NHS Trust, Leeds Vascular Institute; University of Leeds, Clinical Trials Research Unit<br>McGinnis, Elizabeth; Leeds Teaching Hospitals NHS Trust<br>Longo, Roberto; University of Leeds, Academic Unit of Health Economics<br>Game, Frances; National Health Service, Department of Diabetes<br>Bojke, Chris; University of Leeds, Academic Unit of Health Economics<br>Chadwick, Paul; College of Podiatry<br>Chandrasekar, Akila; NHS Blood and Transplant, Tissue Services<br>Chetter, IC; University of Hull<br>Collier, Howard; University of Leeds, Clinical Trials Research Unit<br>Fernandez, Catherine; University of Leeds Faculty of Medicine and Health, Clinical Trials Research Unit<br>Homer-Vanniasinkam, Shervanthi; Leeds Teaching Hospitals NHS Trust<br>Jude, Edward; Tameside General Hospital, Diabetes Centre<br>Leigh, Richard; Royal Free London NHS Foundation Trust<br>Lomas, Richard; NHS Blood and Transplant, Tissue Services<br>Vowden, Peter; Bradford Teaching Hospitals NHS Foundation Trust,<br>Wason, James; Medical Research Council, Biostatistics Unit<br>Sharples, Linda; London School of Hygiene and Tropical Medicine, Medical Statistics<br>Russell, David; Leeds Teaching Hospitals NHS Trust, Leeds Vascular Institute; University of Leeds Leeds Institute of Genetics Health and Therapeutics,
Keywords:	Diabetic foot < DIABETES & ENDOCRINOLOGY, STATISTICS & RESEARCH METHODS, WOUND MANAGEMENT, Clinical trials < THERAPEUTICS

SCHOLARONE™
Manuscripts

Multiple Interventions for Diabetic Foot Ulcer Treatment Trial (MIDFUT): Study Protocol for a randomised controlled trial

Brown, Sarah[1]; Nixon, Jane[1]; Ransom, Myka[1]; Gilberts, Rachael[1]; Dewhirst, Nikki[1,2]; McGinnis, Elizabeth[1,2]; Longo, Roberto[1]; Game, Frances[3]; Bojke, Chris[1]; Chadwick, Paul[4]; Chandrasekar, Akila[5]; Chetter, Ian[6]; Collier, Howard[1]; Fernandez, Catherine[1]; Homer-Vanniasinkam, Shervanthi[2]; Jude, Edward[7]; Leigh, Richard[8]; Lomas, Richard[5]; Vowden, Peter[9]; Wason, James[10,12]; Sharples, Linda[11]; Russell, David[1,2];

[1]University of Leeds, UK; [2]Leeds Teaching Hospitals NHS Trust, UK; [3]Derby Teaching Hospitals NHS Foundation Trust, UK; [4]College of Podiatry, London, UK; [5]NHS Blood and Transplant, Liverpool, UK; [6]University of Hull, UK; [7]Tameside General Hospital, Manchester, UK; [8]Royal Free London NHS Foundation Trust, UK; [9]Bradford Teaching Hospitals NHS Foundation Trust, UK; [10]University of Cambridge, UK; [11]London School of Hygiene and Tropical Medicine, UK; [12] University of Newcastle, UK.

Corresponding author:
Miss Rachael Gilberts
Clinical Trials Research Unit
University of Leeds
Leeds
LS29JT
Email: R.M.Gilberts@leeds.ac.uk
Tel: 0113 343 1724
Fax: 0113 343 37985

Keywords: Diabetic Foot, Diabetes & Endocrinology, Wound Management, Statistics & Research Methods, Clinical Trials

Word count: 7,762

Abstract
Introduction: Diabetes affects more than 425 million people worldwide with a lifetime risk of diabetic foot ulcer (DFU) of up to 25%. Management includes wound debridement, wound dressings, off-loading, treatment of infection and ischaemia, optimising glycaemic control; use of advanced adjuvant therapies is limited by high cost and lack of robust evidence.

Methods and Analysis: A multicentre, seamless Phase II/III, open, parallel group, multi-arm multi-stage randomised controlled trial in patients with a hard-to-heal DFU, with blinded outcome assessment. A maximum of 447 participants will be randomised (245 participants in Phase II and 202 participants in Phase III).

The Phase II primary objective will determine the efficacy of treatment strategies including hydrosurgical debridement +/- decellularised dermal allograft, or the combination with negative pressure wound therapy, as an adjunct to treatment as usual (TAU), compared to TAU alone, with patients randomised in a 1:1:1:2 allocation. The outcome is achieving at least 50% reduction in index ulcer area at 4 weeks post randomisation.

The Phase III primary objective will determine whether one treatment strategy, continued from Phase II, reduces time to healing of the index ulcer compared with TAU alone, with participants randomised in a 1:1 allocation. Secondary objectives will compare healing status of the index ulcer, infection rate, re-ulceration, quality of life, cost-effectiveness and incidence of adverse events over 52 weeks post randomisation.

For peer review only

Phase II and III primary endpoint analysis will be conducted using a mixed-effects logistic regression model and Cox Proportional Hazards regression respectively.

A within-trial economic evaluation will be undertaken; the primary economic analysis will be a cost-utility analysis presenting ICERs for each treatment strategy in rank order of effectiveness, with effects expressed as QALYs.

The trial has pre-defined progression criteria for the selection of one treatment strategy into Phase III based on efficacy, safety and costs at 4 weeks.

Trial registration: ISRCTN64926597; Registered on 06/06/2017.

Ethics and Dissemination: Ethics approval has been granted by NRES Committee Yorkshire & The Humber - Bradford Leeds Research Ethics Committee; approved 26th April 2017; (REC reference: 17/YH/0055).

The datasets during and/or analysed during the current study will be available from the corresponding author on reasonable request.

Article Summary

Strengths and Limitations of this study:

- The multi-arm multi-stage design will allow early evaluation of multiple treatment strategies in a Phase II/III design, stopping treatments which fail to demonstrate sufficient improvement, evaluating only those showing greatest efficacy in a Phase III trial;
- Comparison of multiple treatment strategies to a shared control group, thus requiring fewer participants compared to conventional trial designs;
- Clear pre-defined progression criteria for the selection of the treatment strategy into Phase III;
- Pragmatic in the identification of patients with hard to heal ulcers;
- The target sample size allows only one treatment strategy to be taken forward into Phase III.

For peer review only

Introduction

Diabetes currently affects more than 425 million people worldwide (1), and this is expected to increase to 629 million by 2045 (2). A total of 21-30% of patients with diabetes develop peripheral neuropathy or lose sensation in their feet (3, 4) and extrapolation from incidence studies suggests that lifetime incidence of diabetic foot ulcer (DFU) may be as high as 25% (5). More than 50% of DFUs become infected, requiring hospitalisation, and 20% of infections result in amputation (6), contributing approximately 80% of non-traumatic amputations performed in the developed world (7).

In the UK, diabetes affects 4.5 million people (8) with approximately 64,000 having a DFU at any one time (9). In 2014-15 NHS England spent an estimated £1 billion on DFU treatment (10). This does not take into account the costs imposed on the public sector and society as a whole through working days lost, reductions in tax revenue, increases in benefit payments and social care resources. Furthermore, DFUs have a major impact upon patient health related quality of life (HRQoL), including impaired physical function, mental wellbeing and social interaction (11) .

Management of DFUs comprises provision of NICE recommended best 'treatment as usual' (TAU) through multi-disciplinary team (MDT) DFU clinics (podiatrists, diabetologists, vascular surgeons etc.) and concomitant treatment strategies including: optimising glycaemic control, sharp non-surgical debridement, dressing application, off-loading, treatment of infection and ischaemia (12). There are a number of advanced/adjuvant therapies but their use is limited by high unit cost and an absence of robust evidence.

Despite implementation of MDT care, a national audit of over 33,000 ulcers reports 48.3% remain unhealed at 12 weeks (13). Of those patients with less than the median reduction in DFU area at 4 weeks (<53% reduction), only 9% went onto heal at 12 weeks (14), whilst those in the top half for healing at 4 weeks (≥53% reduction) had a higher probability of healing of 58% at 12 weeks. Delayed healing increases the probability of adverse sequelae including infection and amputation(6). Drivers of the cost of care change with the need for hospitalisation: in a recent study community nurse visits accounted for 65% of total costs for healed and unhealed wounds managed in the outpatient setting, whereas 65% of costs in patients having amputation were incurred in secondary care (15). Thus implementation of adjuvant therapies, which are often more costly, is more likely to be cost effective in those patients identified as "hard to heal" (failing to decrease by >50% at 4 weeks), whereas those DFUs reaching >50% healing at 4 weeks are likely to heal without the need for more expensive interventions. Those DFU's reported as unhealed at 12 weeks in the 2019 UK National Diabetic Foot Audit (13) may have benefitted from such therapies.

Establishing efficacious adjuvant therapies for use in non-healing wounds is a priority to improve healing rates and HRQoL, and reduce the risk of morbidity and cost. There is a paucity of high quality trials assessing adjuvant wound therapies in DFUs and NICE guideline NG19 and others have highlighted the need for randomised controlled trials (RCTs) of negative pressure wound therapy (NPWT) and other adjuvant therapies (12, 16). Technological advances in three adjuvant therapies mean they are now available for routine clinic use.

1) NPWT is available in a small portable pump which doesn't restrict patient movement.
2) Sufficient surgical debridement which changes a chronic wound biology to an acute wound can now be undertaken using hydrosurgical debridement (HD) under local anaesthetic in clinic, enhancing patient experience and reducing costs by avoiding additional hospital visits for day-case surgery. This also allows wound bed preparation to a "graft ready state" for advanced wound adjuncts, a state which cannot be achieved by less aggressive debridement with wound debridement pads such as Debrisoft®. HD has been shown to be as effective as operating theatre surgical debridement in wound healing outcomes (17).
3) Decellularised dermal allograft (DCD), has been used in the USA for treatment of DFU, with improved healing compared to standard care (18, 19) but cost has been prohibitive in the UK. A novel DCD, prepared from skin donated by voluntary UK deceased donors has recently

For peer review only - http://bmjopen.bmj.com/site/about/guidelines.xhtml

been developed within the NHS, is approved by the Human Tissue Authority (HTA) and available for use in the UK. DCD is prepared and supplied by NHS Blood and Transplant (a Department of Health Special Health Authority). However, the application of DCD requires surgical debridement to a 'graft ready wound bed' and it is not known whether the surgical debridement alone leads to improved healing in this setting.

Performing multiple RCTs to assess each intervention individually would be time consuming and expensive. Moreover, these therapies are often used in combination. The MIDFUT trial utilises an efficient, informative and ethical, adaptive, multi-arm, multi-stage (MAMS) design (20). This involves early evaluation of combinations of the candidate interventions in a Phase II/III design, stopping recruitment to treatment strategies which fail to demonstrate sufficient improvements in DFU healing, using an intermediate endpoint at 4 weeks post randomisation, and evaluating only one treatment strategy showing greatest efficacy in a Phase III trial.

The evidence for adjuvant therapies for DFU treatment was reviewed in NICE guideline NG19 (12), which concluded that the quality of trials was poor, due to binary and early endpoints and small sample sizes. It recommended that future trials are sufficiently powered, with outcomes including time to healing, incidence and extent of amputation (major or minor), ulcer recurrence, HRQOL, adverse events, hospital admissions and length of stay. These findings were supported by a review by the International Working Group on the Diabetic Foot (21).

In summary, quality data on the outcome of adjuvant therapies for DFU is rare. A 30% increase in prevalence of disease is anticipated by 2035, which will add to the already substantial costs of treating DFUs, in a time of global fiscal uncertainty. With international guidance advocating the need for robust RCTs in this area (21), and technical advancements in three adjuvant therapy options (HD, DCD and NPWT), an RCT to compare treatment strategies is timely.

Objectives

In Phase II, the aim is to identify the most promising of the three treatment strategies compared to treatment as usual (TAU) using short-term efficacy and in Phase III, to investigate the clinical and cost effectiveness of one treatment strategy from Phase II compared to TAU in the treatment of patients with hard to heal DFUs.

Phase II Primary Objective

To determine the efficacy of the treatment strategies,

1) TAU + HD alone
2) TAU + HD + DCD
3) TAU + HD + NWPT + DCD

compared to TAU alone using the short-term intermediate outcome of achieving at least 50% reduction in index ulcer area at 4 weeks post randomisation (Phase II primary endpoint).

Phase III Primary Objective

To determine whether one treatment strategy continued from Phase II, as an adjunct to TAU reduces time to healing of the index ulcer compared with TAU alone.

The primary endpoint is time *to healing of the index ulcer* from randomisation to the date the index ulcer is confirmed as healed at the first confirmation visit conducted by the blinded assessor (providing the index ulcer is confirmed as healed at a second clinical assessment two weeks later).

Phase III Secondary Objectives:

For peer review only

To compare one treatment strategy as an adjunct to TAU, continued from Phase II, with TAU alone for:

- healing status of the index ulcer at 12, 20 and 52 weeks
- rate of ulcer infection in the foot of the index ulcer over 52 weeks post randomisation
- incidence of re-ulceration following healing of index ulcer over 52 weeks post randomisation
- quality of life using Diabetic Foot Ulcer Scale Short Form (DFS-SF) and EuroQol - five dimensions (EQ-5D-5L) over 52 weeks post randomisation
- incidence of adverse events (including amputation, infection in any ulcer on the foot of the index ulcer and hospital admission) over 52 weeks post randomisation
- cost effectiveness over 52 weeks

Phase III Exploratory Objective:
To explore factors prognostic of ulcer healing

Methods

The MIDFUT trial is a multi-centre, seamless Phase II/III, open, parallel group, multi-arm multi-stage (MAMS) randomised controlled trial (RCT) in patients with a hard to heal DFU, with blinded outcome assessment.

The MAMS trial design will allow an early evaluation of three candidate treatment strategies in a Phase II/III design. Randomisation to treatment strategies that fail to demonstrate sufficient improvement in index ulcer healing at the end of Phase II will be stopped. Only one treatment strategy showing greatest early efficacy will undergo clinical and cost-effectiveness assessment in Phase III (see Figure 1 for a schematic of the design).

Three treatment strategies will be compared to TAU in Phase II. Treatment strategies for which the estimated proportion of responders is less than 10% higher than the proportion of responders on TAU (absolute difference) will be dropped at the end of Phase II (response defined as achieving at least 50% reduction in wound area of the index ulcer). A one treatment strategy and TAU will be evaluated in Phase III. If more than one treatment strategy shows a sufficient response in Phase II, then the decision on which treatment strategy to evaluate in Phase III will consider information on the safety profile and costs of the treatment strategies up to 4 weeks post randomisation.

A maximum of 447 participants will be recruited, 245 participants in Phase II and at most 220 participants in Phase III. Recruitment at centres will continue without interruption between Phase II and Phase III.

All participants from Phases II and III will be followed up at weeks 1, 2, 4, 8, 12, 20 and 52 post-randomisation (including those where healing of the index ulcer has been confirmed), or week 54 where healing of the index ulcer is first reported at week 52 (see Figure 2).

The trial includes a 9-month internal pilot phase in Phase II to evaluate the feasibility of recruitment and therefore delivery of the trial.

Moreover, a Study Within a Trial (SWaT) will be included to determine the extent of agreement in the assessment of healing between central blinded photography review and the clinical assessment of healing.

An interim analysis will be conducted after 220 patients have reached 52 weeks post-randomisation to re-estimate the overall loss to follow-up rate and the final sample size. The review will be conducted in a blinded manner.

For peer review only

Recruitment/Consent

The trial will be conducted in secondary care and community clinics that provide a MDT-DFU service (which includes as a minimum a clinician trained in each trial intervention, podiatrist, diabetologist, vascular surgeon and orthotist).

Patients under the care of the MDT-DFU out-patient clinics, with a current DFU, surgical debridement wound or minor amputation wound, will be assessed for eligibility in accordance with the criteria in Table 1.

Potentially eligible patients will receive a verbal explanation of the study and a Patient Information Leaflet (PIL) by the attending clinical/research team. Strategies to encourage recruitment include:

- posters and/or leaflets in clinic waiting areas and other appropriate locations,
- letter and PIL sent to patients with their out-patient appointment letter,
- study included on relevant websites and research databases that can be accessed by members of the public,
- ethically approved tweets on Twitter.

Following information provision, patients will have as long as they need to consider participation and to discuss the study with their family and other healthcare professionals before consent to participate in the study is requested.

Assenting patients will be invited to provide informed consent and complete an eligibility assessment. Full informed consent will be obtained for all participants prior to the participant undergoing procedures that are specifically for the purposes of the study and are not part of TAU at the participating centre.

Witnessed consent by a representative who is independent of the trial will be available where relevant.

Patients who provide written/witnessed verbal informed consent, but subsequently lose capacity will be withdrawn from the trial.

Non-randomised patients

Participating research sites will complete a log of all patients presenting with a DFU and considered for the trial, but not recruited. Anonymised information to be collected includes age, sex, ethnicity, reason not eligible or reason declined participation.

Randomisation

Following confirmation of eligibility, consent and completion of baseline assessments, participants will be randomised. In Phase II, randomisation will be in a 1:1:1:2 allocation ratio to the three treatment strategies and TAU group respectively, as an approximation to Dunnett's recommendation (22). In Phase III, randomisation will be in a 1:1 allocation ratio to one treatment strategy and TAU. Randomisation in both phases will use a minimisation algorithm, incorporating a random element, via a central 24-hour automated telephone or internet randomisation system, based at the Leeds Clinical Trials Research Unit (CTRU). The dynamic allocation method will ensure the groups are well balanced for:

- Centre
- Aetiology (neuropathic or neuro-ischaemic)
- Index ulcer duration (<6 months, ≥ 6 months)
- Anatomical site (forefoot, mid/hindfoot)
- Presentation (DFU, surgical debridement wound, open minor amputation)

For peer review only

In addition, at the time of randomisation, 25% of participants will be randomly selected to have photographs of the index ulcer taken, if it is unhealed, at weeks 12, 20 and 52, for central blinded review.

Blinding

Due to the nature of the treatment strategies it is not possible to blind participants, the clinicians or research team to the treatment group allocation. However, primary outcome assessments will be conducted by an independent clinical assessor with no knowledge of treatment allocation. To mitigate risk of assessment bias the blinded assessor will also have no access to participant notes or trial Case Report Forms (CRFs). Blinding will be maintained when tracings and photographs at week 4, and confirmation of the index ulcer healing assessments, are returned to the CTRU (e.g. through separate mail or independent clerical staff).

For the Phase II primary outcome and Phase III exploratory objective, a blinded assessor at each site will complete an acetate tracing and take a 2D digital photograph of the index ulcer at week 4. Measurements will be obtained from the index ulcer tracing using 'Image J' software (23) by a member of the CTRU team who is independent of the research teams at recruiting sites and blind to treatment allocation. A photograph of the index ulcer will be taken as a back-up in the event that a tracing cannot be taken or is of insufficient quality to determine the index ulcer outline.

For the Phase III primary endpoint, all participants recruited to both Phase II and III will also have a photograph taken of the reported healed index ulcer by the blinded assessor within 3 days of healing being reported and two weeks later as a confirmation of healing, which will undergo blinded central review.

Photographs taken of healed and of unhealed index ulcers for randomly selected participants will be submitted for central blinded photography review by clinical members of the Trial Management Group who will not be aware of the participant's identity, treatment group or time point at which the photograph was taken.

Interventions

All randomised treatment strategies will be applied to the index ulcer as a "once only intervention" on the day of randomisation in the MDT-DFU service clinic, with the exception of NPWT which will be applied until the 2-week visit. Treatment of any other ulcers will continue as per the treating clinician decision.

At baseline, randomisation and each follow-up visit all participants will receive TAU. At the randomisation treatment visit, the participant will be randomised to receive the treatment strategy specific to the arm of the trial for the index ulcer. This will include one or more of the following:

Treatment as usual (TAU)

Participants will receive the minimum standard care provided by the recruiting centre. This will be in line with NICE guidelines (12) and is likely to include attendance at the MDT-DFU service clinic(s) at least fortnightly until healing is confirmed for wound assessment, sharp non-surgical debridement of callous/non-viable tissue, review of off-loading and to optimise diabetes and wound assessment as required, including community services visits, typically 1 to 2 times weekly. In line with NICE guidelines, use of removable below-knee walking device or removable cast walker will be encouraged. Wound dressing changes will be performed between clinic visits according to local policies.

Hydrosurgical Debridement (HD)

Hydrosurgical Debridement (HD), a one-off procedure on the day of randomisation, applies saline at

For peer review only

high pressure via a pump through a hand piece. This has an operating window located at the instrument's distal tip. During operation the flow of pressurised saline creates a local vacuum. As the operating window of the handset is passed over the tissue, non-viable material and debris are removed. The ulcer bed is debrided to healthy bleeding tissue which may require local anaesthetic.

Negative Pressure Wound Therapy (NPWT)

Negative Pressure Wound Therapy (NPWT), applied on the day of randomisation, consists of a foam dressing cut to shape and applied to the wound. An air-tight seal is established with a film dressing; this is then connected to a pump which applies gentle suction to the wound. This allows the removal of fluid from the wound, which is collected in a canister attached to the pump, which is carried by the participant at all times in the bag provided. Alternatively, a self-contained system consisting of a disposable NPWT pump attached to an absorbent adhesive dressing may be used. The dressing is usually changed at least once a week, and the NPWT will be applied for 2 weeks post randomisation.

Decellularised Dermal Allograft (DCD)

Decellularised Dermal Allografts are prepared from split skin grafts obtained from deceased human tissue donors, which are processed and sterilised. Processing retains the normal skin structure, but removes donor cells and cell remnants meaning the graft is not rejected and functions as a permanent tissue replacement. Upon receipt at participating sites, the graft will be stored between 0-40°C until the expiry date stated on the graft label. Prior to application the graft is soaked in a bowl of sterile saline solution for 15 minutes. The graft is then cut to size using sterile scissors and applied directly to the debrided wound bed, epidermal side upwards. Following application, the ulcer is covered with a non-adherent contact layer and a secondary bolster dressing or NPWT (as per randomisation). In those DFUs allocated to DCD, the wound bed is not debrided for 4 months post-treatment, unless clinically indicated, although debridement of wound edge and surrounding tissue can continue as per TAU.

Assessments/data collection and follow-up

Baseline Assessment

Participant demographics including date of birth, gender, ethnicity, NHS number, and site of the index ulcer will be recorded.

Clinical history will be recorded including smoking history, duration and type of diabetes, number of ulcers and index ulcer characteristics e.g. first or recurrent ulcer, aetiology, existing wound therapies and SINBAD classification (24). Initial index ulcer area tracing (using acetate) will also be obtained. Participants will be asked to complete quality of life questionnaires: Diabetic Foot Scale –Short Form (DFS-SF) and the EuroQoL-5D 5 level (EQ-5D-5L).

Randomisation and application of the treatment strategy will take place after baseline assessments and questionnaires have been completed, on the same day.

Information collected post treatment will include details of the treatment strategy applied to the index ulcer and to any other ulcers on the foot of the index ulcer, and expected adverse events (AEs) and serious adverse events (SAEs). Post HD debridement index ulcer area acetate tracing and photographs will be obtained.

Follow-up Assessments

At a routine clinic assessment at week 1 and at week 2, 4, 12, 20 and 52 post-randomisation the following assessments will be conducted by a member of the clinical research team (clinician, clinical research nurse or registered healthcare professional): healing status of the index ulcer, episodes of infection in the foot of the index ulcer (IDSA criteria), revascularisation of the index limb, index ulcer treatments and expected AEs or SAEs. In addition, at weeks 1 and 2 post-randomisation, an assessment of compliance with NPWT and DCD (where applicable), and at weeks 2 to 52 post-

For peer review only

randomisation an assessment of re-ulceration of the index ulcer will be conducted. At week 2 and week 4 post randomisation, an acetate tracing and photograph of the index ulcer post sharp non-surgical debridement (where clinically indicated) will be taken; at week 4 this is conducted by a blinded assessor (clinician, research nurse or registered healthcare professional).

Patient questionnaires (DFS-SF, EQ-5D-5L and Health Resource Utilisation (HRU)) will be completed at weeks 4, 12, 20 and 52 post-randomisation; the HRU questionnaire will also be completed at week 8 post-randomisation.

Healing and Re-ulceration Assessments

Healing is defined as complete closure of the ulcer: 100% re-epithelialisation of the wound surface with the absence of drainage, confirmed by blinded assessment of index ulcer healing status at two consecutive assessments two weeks apart (25).

Healing of the index ulcer will be reported in one of the following scenarios;

- By the Research Nurse/Registered Healthcare Professional at a research visit.
- During the participant's routine appointment at the MDT-DFU service clinic, podiatry clinic, GP practice nurse and/or at home by district nurses as per treatment as usual
- Patient self-reporting to the research team or to the attending clinical team in between routine appointments who will then inform the research team.

The attending clinical team will contact the research team to report the date the index ulcer was first noted as healed, who will then arrange an initial visit within 3 days of healing of the index ulcer first being reported and a 2 week follow-up visit (+/- 3 days) with the blinded assessor to assess index ulcer healing status and conduct photography.

Re-ulceration is defined as recurrence of a full thickness break in the epithelium at the same location as the index ulcer (26). Re-ulceration of the index ulcer will be established either by participant self-referral to the research team, at a routine clinic or research appointment or by continuous screening of new referrals to the MDT-DFU service clinic where participants will be flagged to the research team by the attending clinical team. Re-ulceration of the index ulcer will be confirmed by a blinded assessor, within 7 days of re-ulceration being reported, with reference to the photograph of the foot taken at the randomisation visit, photography undertaken and the date of re-ulceration of the index ulcer recorded.

Sample size

The planned maximum sample size is 447 patients, 245 patients in Phase II and 202 patients in Phase III. The apportionment of participants to Phase II and Phase III was estimated using a series of simulation studies.

In Phase II, 49 patients per treatment strategy arm and 98 patients in the TAU arm will be recruited. The target effect size in Phase II is an absolute increase of 25% in the proportion of patients achieving at least a 50% reduction in wound area by 4 weeks post randomisation, assuming 39% reach at least a 50% reduction by week 4 in the TAU arm (local audit data) and 64% achieve this outcome in the treatment strategy arms.

An additional 101 patients will be recruited into each arm evaluated in Phase III, corresponding to a total (Phase II and III combined) of 150 in the remaining treatment strategy group and 199 in the TAU arm (total of 349 patients for evaluation in Phase III).

The minimum clinically important effect size in Phase III is a hazard ratio of 1.5, assuming a median time to healing of 21 weeks for the TAU arm (local audit data) and 14 weeks for the treatment strategy arms (18, 27-30) and 18.0% and 7.6% ***un***healed at 52 weeks in the TAU and treatment strategy group respectively (assuming exponential distribution for time to healing).

For peer review only

Several scenarios for the power of the trial have been considered. In all cases a 10% loss to follow-up by 4 weeks and 25% loss to follow up by 52 weeks is assumed. In the case where there is a single effective treatment strategy arm, the design has 83% power to recommend a truly effective treatment strategy (i.e. for it to progress from Phase II and for a significant result found at Phase III). A treatment strategy group that progresses to Phase III and which is significantly better than TAU at the 2-sided 4% significance level (to control the family wise error rate at 5%) on the time to healing endpoint will be declared clinically effective.

A formal sample size review will be conducted at 52 weeks, after 220 patients have been recruited, to re-estimate the proportion of patients lost to follow-up by 52 weeks post randomisation and the final sample size. The review will allow the overall loss to follow-up to be estimated to a minimum precision ±5.7% (corresponding to half width of the 95%CI), assuming a maximum loss to follow-up of 25%.

Progression criteria for Phase III

The minimum criterion for taking treatment strategies forward into Phase III will be defined as at least a 10% increase in the probability of achieving ≥50% reduction in index ulcer area at 4 weeks post randomisation above that observed for TAU, corresponding to the minimum clinically important difference (clinical opinion). If more than one treatment strategy passes this threshold at Phase II then the selection criteria will be based on a combination of efficacy, safety profile and cost of treatment strategies up to 4 weeks post-randomisation. The progression criteria are provided in further detail in Table 2.

The trial will stop on the basis of futility if no treatment strategy continues into Phase II. This will be non-binding to allow the Data Monitoring and Ethics Committee (DMEC) to make more nuanced recommendations and the Trial Steering Committee (TSC) the final decision on whether or not to stop the trial.

Statistical analysis

Statistical analysis plans for Phase II and Phase III final analyses will be finalised and signed off before any data analyses are conducted.

The complete case population will be used for the analysis of the Phase II endpoint under the assumption that data are missing at random (MAR) (31). A sensitivity analysis will be considered if there is differential missing endpoint data observed across treatment arms.

Phase III analyses will use Intention to Treat (ITT) whereby patients will be analysed according to randomised treatment group. A per-protocol population will also be defined.

For Phase III endpoint analyses, data from all participants recruited in Phase II and Phase III will be included.

Phase II Primary Endpoint Analysis

Primary analysis

Treatment effects and 95% CI on response at 4 weeks will be estimated from multivariable mixed-effects logistic regression, including the minimisation factors and treatment group as fixed effects and centres as random effects. Simple contrasts for each treatment strategy compared to the TAU arm will be used.

Phase II secondary endpoint analysis

For peer review only

AEs and SAEs that are classified as expected and related to DFUs and trial treatment strategies, or 'related and unexpected SAEs' (RUSAEs) will be summarised by treatment group.

Mean per-patient costs of treatment, health care use and total resource use, together with a measure of variance will be reported by treatment group.

Phase III Primary Endpoint Analysis

Primary analysis

The hazard ratios for the Phase III endpoint will be estimated using Cox Proportional Hazards regression with covariates for the minimisation factors, treatment arm (fixed effects) and centre (random effects) and stratification for the phase in which the patient was recruited.

Phase III secondary endpoint analysis

Similar regression-based analyses will be used for other secondary endpoints. Cumulative incidence of healing at 12, 20 and 52 weeks post randomisation will be obtained from the primary endpoint analysis model. A Poisson-Gamma regression model will be fitted to infection status over time. A Cox Proportional Hazard's regression model will be fitted to time to re-ulceration of the index ulcer on those patients where healing of the index ulcer is confirmed. A repeated measures, random coefficients, linear regression model will be fitted to the DFS-SF score over time.

All adverse events and serious adverse events, including amputations and admissions to hospital, will be recorded and summarised by treatment strategy received. Expected treatment-related adverse events (AEs) include: pain, bleeding and infection from hydrosurgical debridement; bleeding, infection and skin irritation/breakdown from NPWT; seroma and allergic reaction from DCD.

Exploratory Analyses

Sensitivity analyses

For all analyses using the Cox Proportional Hazard's model, the assumption of independence of the distribution of time to healing /recurrence and time to other events, i.e. amputation and death will be assessed and alternative models considered if there are sufficient competing risks.

A multivariable Cox Proportional Hazards regression model will be fitted to explore risk factors predictive of time to healing.

Economic evaluation

A within-trial economic evaluation will be undertaken at week 52 post-randomisation. The proposed secondary endpoints and methods for the economic evaluation follow the reference case set out by NICE (32). The primary economic analysis will be a cost-utility analysis presenting incremental cost-effectiveness ratios (ICER) for each treatment strategy in rank order of effectiveness, with effects expressed in terms of quality-adjusted life years (QALY). An NHS and Personal Social Services (PSS) perspective for costs will be adopted. Costs and effects for each treatment strategy will be calculated for the trial follow-up period of 52 weeks.

Health resource use questionnaires will collect information on NHS and personal social care use in line with NICE guidelines (32). This will include primary, secondary, and community resource use. Unit cost data will be obtained from national databases such as the British National Formulary and Personal Social Services Research Unit (PSSRU) Costs of Health and Social Care.

Treatment costs include the cost of delivering each strategy (mainly given by person-time of health-care professionals) and the cost of the necessary equipment. The scope of resources considered includes the direct healthcare costs incurred for necessary patient care and excludes resources

For peer review only

driven by the study protocol (e.g., routine clinics will be included, whilst research visits that are just for checking for re-ulceration are excluded; also, the cost of photography and visit time for collecting data for study purposes will be excluded). To cost the treatment strategies, data on average duration of appointments for delivering the treatment strategy will be collected.

Incremental cost-effectiveness ratios, Incremental Net Monetary Benefit (INMB) and Incremental Net Health Benefit (INHB) statistics will be computed. The National Institute for Health and Care Excellence (NICE) considers a cost per QALY within the range of £20,000-£30,000 to be acceptable (32).

Multiple Imputation will be used to address any issues of missing data in the base case analysis on the assumption of MAR. Complete Case analysis will be conducted as a sensitivity analysis.

Probabilistic Sensitivity Analysis (PSA) will be used to assess the impact of sampling uncertainty on the within trial evaluation results. Simulated cost and QALY estimates will be plotted on the cost-effectiveness plane to illustrate the uncertainty surrounding cost-effectiveness estimates (33) and presented as cost-effectiveness acceptability frontier (CEAF) to capture the varying probability of interventions being the most cost-effective over a range of willingness to pay for a QALY thresholds (34)

In addition, alternative scenarios will be explored in the sensitivity analysis to test the robustness of the main trial analysis results including analysis of complete cases only.

Data Management

Data will be monitored for quality and completeness and missing data will be chased up. Data received, including photographs will be stored in a secure database at Leeds CTRU in accordance with the 2018 Data Protection Act and the General Data Protection Regulation (GDPR).

Patient and Public Involvement

The trial was supported at the stage of developing the grant application by the Sheffield Teaching Hospitals Lay Advisory Panel for Diabetes & Endocrinology Research. Their input was central to study design, actively helping to shape discussions and decisions through written feedback and group discussion. In particular they informed decisions on the how frequently patients should be reassessed at the study site; whether patients would be willing to report healing or other outcomes directly to the research team or through their community clinician; agreement to completion of questionnaires, wound tracing and photographs which may be considered a burden; how willing patients will be to take part in the study; the acceptability of each intervention.

The trial has two PPI representatives on the Trial Steering Committee who have provided input into the patient information sheet and other trial documentation intended for use by patients. The PPI representatives also provide input into the design and conduct of the trial at 6 monthly meetings. This high-level involvement in project management aims to ensure patients perspectives are fully integrated in key decisions about the trial, delivery, and interpretation/dissemination of findings.

Discussion

MAMS design:

The chosen MAMS design provides an efficient platform for assessment of several competing interventions, quickly homing in on the treatment strategy with greatest potential to be effective, early dropping of ineffective treatments and allowing assessment of combinations of treatments (20, 35-37). Specific advantages of the MAMS design include comparing multiple treatment arms to a shared control group thereby requiring fewer patients, improved consent/ recruitment rates since patients are more likely to receive an active treatment (37) and common eligibility criteria across trial arms (20).

For peer review only

Moreover, recruitment to all treatment strategies will continue during analysis and reporting of Phase II data to ensure no loss of momentum in recruitment at sites.

Choice of endpoints:
Reduction in ulcer area at 4 weeks was chosen as the primary outcome at Phase II to be consistent with published observational studies (38, 39) and DFU RCTs (40). This intermediate outcome measure provides a means of screening for “emerging evidence” of efficacy, as it occurs earlier and more frequently than the definitive outcome measure and that it is on the causal pathway (41). Thus, it allows the Phase II analysis and decision on treatment selection to take place in a timely manner.

Time to healing, the primary endpoint in Phase III, was chosen as an important outcome measure from both clinical and economic perspectives (30).

Ulcer infection is the most common complication in non-healing ulcers, occurring in more than 50% of DFUs (6). It results in delayed healing, prolonged treatment, increased resource use and increases the risk of a patient requiring a major amputation (42). IDSA criteria is recognised as a gold standard for characterisation of infection in DFUs in many national and international guidelines and will provide a reproducible system for clinical diagnosis and severity stratification (12) (21) (43).

No single patient-reported outcome measure (PROM) has been identified as a “gold standard” for assessing HRQOL in diabetes-related foot disease (12). As a result, both the disease-specific questionnaire, DFS-SF (44) and the preference-based utility measure, EQ-5D-5L (www.euroqol.org) (45), will be completed by patients. The DFS-SF questionnaire has acceptable psychometric properties for measuring quality of life for patients with DFUs. The EQ-5D is a generic instrument and forms part of the NICE reference case for cost per Quality Adjusted Life Years (QALY) analysis.

Blinded assessment of healing
Having a blinded assessment of healing is important in reducing the risk of assessment bias and the trial includes independent, blinded clinical assessment of healing at both the first and confirmation of healing assessments, along with additional blinded review of photography undertaken by clinicians (CI (vascular surgeon) and clinical nurse specialists).

Revision to trial design
Since opening, the trial has undergone a trial re-design. The original design included a fourth treatment strategy in Phase II corresponding to a combination of HD and NPWT as an adjunct to TAU, and also allowed a maximum of two treatment strategies to go forward into Phase III under the same progression criteria. This original design required a maximum sample size of 660 participants, 324 participants recruited in Phase II and 336 in Phase III, under the sample size assumptions. Following a review of treatment strategies which would be considered in clinical practice if shown to be clinically and cost effective, a revised trial design dropped the combination of HD and NPWT arm, which reduced the maximum sample size to 447 patients whilst still ensuring a trial of clinical relevance.

Hard-to-heal ulcers
A registration phase was also included in the original trial design. It is important that the trial includes a patient population with “hard-to-heal” ulcers, reflecting the target population that would be considered for such adjuvant therapies in clinical practice. As there is variability in usual wound area assessment and documentation across recruiting centres, the registration phase included in the original design allowed a consistent approach to assessment of healing over a 4-week run-in period, and thereby considered “best practice” for trials of DFU healing. However, the recently published LeucoPatch trial (46), using an identical registration phase criterion of 50% healing at 4 weeks, reported only 22% healing in the control and 34% healing in the intervention arm, suggesting that this

For peer review only

registration phase criteria is overly selective in the definition of “hard-to-heal”. Early audit of data from a single centre in MIDFUT showed that patients were not recruited to the trial at presentation to an MDT DFU clinic, and had been subjected to an “in house assessment period” prior to registration in the trial. 75% of those failing the trial registration period due to ulcer healing of >50% remained unhealed at 12 weeks, suggesting the trial was excluding a group of ulcers that were in fact “hard-to-heal”. The 2018 UK National Diabetic Foot Care Audit data of 21,000 ulcer episodes reports 49.3% remain unhealed at 12 weeks, with 27.3% unhealed 24 weeks and a further 2.9% recurring. Thus under half of ulcers unhealed at 12 weeks will heal in the subsequent 12 weeks, supporting a second inclusion criterion of ulcers that remain unhealed at a 12-week timepoint (9). A parallel entry route was therefore introduced to allow patients with ulcers of ≥12 weeks duration, that are considered hard-to-heal by the treating MDT team, to proceed directly to randomisation.

Following challenges in recruiting patients and the supporting evidence that a large proportion of patients with hard-to-heal ulcers were being missed, a decision was made to drop the Registration phase for all patients. Instead, included ulcers will have failed to reduce in area by >50% over at least 4 weeks as measured using local measurement techniques. This allows a more pragmatic approach to be taken in identifying patients with hard to heal ulcers by using local wound measurement policies, thereby minimises the risk of missing potentially eligible patients whilst ensuring the trial results are more generalisable to the target patient population.

Adjuvant therapies
It is not anticipated that there will be a rapid change in the technologies investigated in this trial, other than design changes aimed at increasing clinician and patient acceptance, again increasing the potential adoption and generalisability of the trial outcomes.

Trial Status
The first participant was registered on 10th August 2017 and the first participant randomised on 30th October 2017. As of 19th November 2019, 165 participants have been registered and 86 randomised. Recruitment is expected to complete by 31st August 2022. The full trial protocol is available on the NIHR journals library https://www.journalslibrary.nihr.ac.uk/programmes/hta/150877/#/.

Declarations Ethics and Dissemination: Informed consent was provided by participants prior to commencement of their study. Ethics approval has been granted by NRES Committee Yorkshire & The Humber - Bradford Leeds Research Ethics Committee; approved 26th April 2017; (REC reference: 17/YH/0055).

Abbreviations

AE	Adverse Event
CEAF	Cost-Effectiveness Acceptability Frontier
CRF	Case Report Form
DCD	Decellularised Dermal Allograft
DMEC	Data Monitoring and Ethics Committee
DFU	Diabetic Foot Ulcer
DFS-SF	Diabetic Foot Scale-Short Form
eGFR	Estimated glomerular filtration rate
EQ-5D-5L	EuroQoL-5D 5 level
GDPR	General Data Protection Regulation
HbA1c	Haemoglobin A1c
HD	Hydrosurgical Debridement
HRQoL	Health Related Quality of Life
HRU	Health Resource Utilisation

For peer review only

HTA	Human Tissue Act
HTA	Health Technology Assessment
ICER	Incremental Cost Effective Ratio
IDSA	Infectious Diseases Society of America
INHB	Incremental Net Health Benefit
ITT	Intention to Treat
MAMS	Multi Arm Multi Stage
MAR	Missing At Random
MDT	Multidisciplinary team
NMB	Net Monetary Benefit
NHS	National Health Service
NHS BT	National Health Service Blood and Transplant
NICE	National Institute for Health and Care Excellence
NIHR	National Institute for Health Research
NPWT	Negative Pressure Wound Therapy
PROM	Patient Reported Outcome Measure
PSA	Probabilistic Sensitivity Analysis
PIL	Participant Information Leaflet
PSSRU	Personal Social Services Research Unit
QALY	Quality Adjusted Life Years
RCT	Randomised Controlled Trial
REC	Research Ethics Committee
RUSAE	Related Unexpected Serious Adverse Event
SAE	Serious Adverse Event
SWAT	Study Within a Trial
TAU	Treatment as Usual
TS	Treatment Strategy
TSC	Trial Steering Committee
WHO	World Health Organisation

Authors' contributions

DR, as the CI, conceived the trial, provided clinical expertise in the design and conduct of the trial, has overall leadership for the trial; JN provided expertise in the trial design and has scientific oversight; LS and JW provided senior statistical expertise in the trial design and have statistical and methodological oversight of the trial; SB provided statistical input on the trial design and is the supervising statistician, led on drafting the manuscript; MR is responsible for conducting the statistical analysis and reporting of the trial; RG is responsible for the coordination of the trial; CF is responsible for the overall management of the trial; HC is responsible for acquisition and management of the clinical data; ND and EM are Clinical Co-ordinators involving centre set-up support and ongoing monitoring; RL/CB has responsibility for the health economic design considerations and analysis; FG, EM, ND, PC, RL, SHV, IC, PV, EJ provided clinical expertise in the design and conduct of the trial; AC and RL provided expertise in the trial design and DCD. All authors reviewed draft versions and approved the final manuscript.

Funding Statement

This study was funded by the National Institute for Health Research (NIHR) Health Technology Assessment (HTA) Programme (Project: 15/08/77). The views and opinions expressed therein are those of the authors and do not necessarily reflect those of the HTA, NIHR, NHS or Department of Health.

We received product support for Versajet consoles and NPWT pumps from Smith & Nephew

For peer review only - http://bmjopen.bmj.com/site/about/guidelines.xhtml

We also received support for DCD from NHSBT and a £160K research support grant to cover excess treatment costs for consumables.

Competing interests

None: DR, FG, EJ, SHV, PC, JW, LS, RG, ND, EM, RL, MR, HC, CF, JN, SB

AC and RL are employees of NHS BT who provide the DCD.

Acknowledgments

The authors would like to thank members of the Trial Steering Committee: Professor Simon Heller, Dr Jill Cundell, Professor Rob Hinchcliffe, Professor Steven Julious, Ms Brenda Riley, Ms Sonia Ward and in memory of Mr Paul Higgins; and members of the Data Monitoring and Ethics Committee: Mr Matt Sydes, Professor Julie Brittenden, Dr Magnus Löndahl, Mr Robert Davies and Professor Boby Mihaylova.

Licence Statement

I, the Submitting Author has the right to grant and does grant on behalf of all authors of the Work (as defined in the below author licence), an exclusive licence and/or a non-exclusive licence for contributions from authors who are: i) UK Crown employees; ii) where BMJ has agreed a CC-BY licence shall apply, and/or iii) in accordance with the terms applicable for US Federal Government officers or employees acting as part of their official duties; on a worldwide, perpetual, irrevocable, royalty-free basis to BMJ Publishing Group Ltd ("BMJ") its licensees and where the relevant Journal is co-owned by BMJ to the co-owners of the Journal, to publish the Work in BMJ Open and any other BMJ products and to exploit all rights, as set out in our licence.

The Submitting Author accepts and understands that any supply made under these terms is made by BMJ to the Submitting Author unless you are acting as an employee on behalf of your employer or a postgraduate student of an affiliated institution which is paying any applicable article publishing charge ("APC") for Open Access articles. Where the Submitting Author wishes to make the Work available on an Open Access basis (and intends to pay the relevant APC), the terms of reuse of such Open Access shall be governed by a Creative Commons licence – details of these licences and which Creative Commons licence will apply to this Work are set out in our licence referred to above.

For peer review only

References:

1. IDF Diabetes Atlas. IDF Diabetes Altas eighth edition 2017. [Available from: https://www.idf.org/aboutdiabetes/what-is-diabetes/facts-figures.html.
2. IDF Diabetes Atlas. IDF Diabetes Altas sixth edition 2014 Update [6:[Available from: http://www.idf.org/sites/default/files/Atlas-poster-2014_EN.pdf.
3. Abbott CA, Malik RA, van Ross ER, Kulkarni J, Boulton AJ. Prevalence and characteristics of painful diabetic neuropathy in a large community-based diabetic population in the UK. Diabetes care. 2011;34(10):2220-4.
4. Salvotelli L, Stoico V, Perrone F, Cacciatori V, Negri C, Brangani C, et al. Prevalence of neuropathy in type 2 diabetic patients and its association with other diabetes complications: The Verona Diabetic Foot Screening Program. Journal of diabetes and its complications. 2015;29(8):1066-70.
5. Singh N, Armstrong DG, Lipsky BA. Preventing foot ulcers in patients with diabetes. Jama-Journal of the American Medical Association. 2005;293(2):217-28.
6. Lavery LA, Armstrong DG, Wunderlich RP, Mohler MJ, Wendel CS, Lipsky BA. Risk factors for foot infections in individuals with diabetes. Diabetes Care. 2006;29(6):1288-93.
7. Armstrong DG, Lavery LA. Diabetic foot ulcers: Prevention, diagnosis and classification. American Family Physician. 1998;57(6):1325-32.
8. Diabetes UK https://www.diabetes.org.uk/Professionals/Position-statements-reports/Statistics/Diabetes-prevalence-2016/.
9. National Diabetes Foot Care Audit Report 2014-2017. https://files.digital.nhs.uk/pdf/e/9/ndfa-3ar-rep.pdf.
10. Kerr M (2017) Diabetic foot care in England: An economic study. https://www.diabetes.org.uk/Upload/Shared practice/Diabetic footcare in England, An economic case study (January 2017).pdf.
11. Hogg F, Peach G, Price P, Thompson M, Hinchliffe R. Measures of health-related quality of life in diabetes-related foot disease: a systematic review. Diabetologia. 2012;55(3):552-65.
12. NICE NG19. Diabetic foot problems: prevention and management 2015 [Available from: https://www.nice.org.uk/guidance/ng19.
13. National Diabetes Foot Care Audit Report 2015-2018 https://files.digital.nhs.uk/50/8E75BA/NDFA%204AR%20-%20Main%20Report%20v1.0.pdf accessed 5/11/19.
14. Sheehan P, Jones P, Caselli A, Giurini JM, Veves A. Percent change in wound area of diabetic foot ulcers over a 4-week period is a robust predictor of complete healing in a 12-week prospective trial. Diabetes Care. 2003;26(6):1879-82.
15. Guest JF, Fuller GW, Vowden P. Diabetic foot ulcer management in clinical practice in the UK: costs and outcomes. International wound journal. 2018;15(1):43-52.
16. Dumville JC, Hinchliffe RJ, Cullum N, Game F, Stubbs N, Sweeting M, et al. Negative pressure wound therapy for treating foot wounds in people with diabetes mellitus. Cochrane Database of Systematic Reviews. 2013(10).
17. Caputo WJ, Beggs DJ, DeFede JL, Simm L, Dharma H. A prospective randomised controlled clinical trial comparing hydrosurgery debridement with conventional surgical debridement in lower extremity ulcers. International Wound Journal. 2008;5(2):288-94.
18. Brigido SA. The use of an acellular dermal regenerative tissue matrix in the treatment of lower extremity wounds: a prospective 16-week pilot study. International Wound Journal. 2006;3(3):181-7.
19. Reyzelman A, Crews RT, Moore JC, Moore L, Mukker JS, Offutt S, et al. Clinical effectiveness of an acellular dermal regenerative tissue matrix compared to standard wound management in healing diabetic foot ulcers: a prospective, randomised, multicentre study. International Wound Journal. 2009;6(3):196-208.

For peer review only

20. Pallmann P, Bedding AW, Choodari-Oskooei B, Dimairo M, Flight L, Hampson LV, et al. Adaptive designs in clinical trials: why use them, and how to run and report them. BMC Medicine. 2018;16(1):29.
21. Game FL AJ, Attinger C, Hartemann A, Hinchliffe RJ, Löndahl M, Price PE, Jeffcoate WJ on behalf of the International Working Group on the Diabetic Foot (IWGDF). . Effectiveness of interventions to enhance healing of chronic ulcers of the foot in diabetes: a systematic review. Diabetes/Metabolism Research and Reviews. 2016;32(S1):154-68.
22. Dunnett CW. A multiple comparison procedure for comparing several treatments with a control. Journal of the American Statistical Association. 1955;50(272):1096-121.
23. Schneider CA, Rasband WS, Eliceiri KW. NIH Image to ImageJ: 25 years of image analysis. Nature methods. 2012;9(7):671-5.
24. Ince P, Abbas ZG, Lutale JK, Basit A, Ali SM, Chohan F, et al. Use of the SINBAD classification system and score in comparing outcome of foot ulcer management on three continents. Diabetes care. 2008;31(5):964-7.
25. J Jeffcoate W, Bus S, Game F, J Hinchliffe R, Price P, Schaper N. Reporting standards of studies and papers on the prevention and management of foot ulcers in diabetes: required details and markers of good quality2016.
26. Peters EJ, Armstrong DG, Lavery LA. Risk factors for recurrent diabetic foot ulcers: site matters. Diabetes Care. 2007;30(8):2077-9.
27. Edmonds M, European Australian A. Apligraf in the Treatment of Neuropathic Diabetic Foot Ulcers. International Journal of Lower Extremity Wounds. 2009;8(1):11-8.
28. Veves A, Falanga V, Armstrong DG, Sabolinski ML, Apligraf Diabetic Foot Ulcer S. Graftskin, a human skin equivalent, is effective in the management of noninfected neuropathic diabetic foot ulcers - A prospective randomized multicenter clinical trial. Diabetes Care. 2001;24(2):290-5.
29. Veves A, Sheehan P, Pham HT, Promogran Diabetic Foot Ulcer S. A randomized, controlled trial of promogran (a collagen/oxidized regenerated cellulose dressing) vs standard treatment in the management of diabetic foot ulcers. Archives of Surgery. 2002;137(7):822-7.
30. Gottrup F, Cullen BM, Karlsmark T, Bischoff-Mikkelsen M, Nisbet L, Gibson MC. Randomized controlled trial on collagen/oxidized regenerated cellulose/silver treatment. Wound Repair and Regeneration. 2013;21(2):216-25.
31. White IR, Carpenter J, Horton NJ. Including all individuals is not enough: lessons for intention-to-treat analysis. Clinical trials. 2012;9(4):396-407.
32. NICE. Guide to the methods of technology appraisal 2013 [Available from: https://www.nice.org.uk/process/pmg9/chapter/foreword#discounting-2.
33. O'Brien BJ, Briggs AH. Analysis of uncertainty in health care cost-effectiveness studies: an introduction to statistical issues and methods. Statistical Methods in Medical Research. 2002;11(6):455-68.
34. Fenwick E, O'Brien BJ, Briggs A. Cost-effectiveness acceptability curves - facts, fallacies and frequently asked questions. Health Economics. 2004;13(5):405-15.
35. Wason JMS, Jaki T. Optimal design of multi-arm multi-stage trials. Statistics in Medicine. 2012;31(30):4269-79.
36. Schmidli H, Bretz F, Racine A, Maurer W. Confirmatory Seamless Phase II/III Clinical Trials with Hypotheses Selection at Interim: Applications and Practical Considerations. Biometrical Journal. 2006;48(4):635-43.
37. Parmar MKB, Carpenter J, Sydes MR. More multiarm randomised trials of superiority are needed. The Lancet. 2014;384(9940):283-4.
38. Margolis DJ, Gelfand JM, Hoffstad O, Berlin JA. Surrogate end points for the treatment of diabetic neuropathic foot ulcers. Diabetes care. 2003;26(6):1696-700.
39. Snyder RJ, Cardinal M, Dauphinee DM, Stavosky J. A Post-hoc Analysis of Reduction in Diabetic Foot Ulcer Size at 4 Weeks as a Predictor of Healing by 12 Weeks. Ostomy Wound Management. 2010;56(3):44-+.

For peer review only

40. Game F, Jeffcoate W, Tarnow L, Day F, Fitzsimmons D, Jacobsen J. The LeucoPatch® system in the management of hard-to-heal diabetic foot ulcers: study protocol for a randomised controlled trial. Trials. 2017;18(1):469.
41. Sydes MR, Parmar MK, James ND, Clarke NW, Dearnaley DP, Mason MD, et al. Issues in applying multi-arm multi-stage methodology to a clinical trial in prostate cancer: the MRC STAMPEDE trial. Trials. 2009;10(1):39.
42. Pickwell K, Siersma V, Kars M, Apelqvist J, Bakker K, Edmonds M, et al. Predictors of lower-extremity amputation in patients with an infected diabetic foot ulcer. Diabetes Care. 2015;38(5):852-7.
43. Lipsky BA, Aragón-Sánchez J, Diggle M, Embil J, Kono S, Lavery L, et al. IWGDF guidance on the diagnosis and management of foot infections in persons with diabetes. Diabetes/metabolism research and reviews. 2016;32:45-74.
44. Bann CM, Fehnel SE, Gagnon DD. Development and validation of the Diabetic Foot Ulcer Scale-short form (DFS-SF). Pharmacoeconomics. 2003;21(17):1277-90.
45. EuroQol Group. What is EQ-5D 2015 [Available from: www.euroqol.org/eq-5d/what-is-eq-5d.
46. Game F, Jeffcoate W, Tarnow L, Jacobsen JL, Whitham DJ, Harrison EF, et al. LeucoPatch system for the management of hard-to-heal diabetic foot ulcers in the UK, Denmark, and Sweden: an observer-masked, randomised controlled trial. The lancet Diabetes & endocrinology. 2018;6(11):870-8.
47. World Health Organisation. Definition and Diagnosis of Diabetes Mellitus and Intermediate Hyperglycemia 2006 [Available from: http://apps.who.int/iris/bitstream/10665/43588/1/9241594934_eng.pdf.
48. Lipsky BA, Berendt AR, Cornia PB, Pile JC, Peters EJG, Armstrong DG, et al. 2012 Infectious Diseases Society of America Clinical Practice Guideline for the Diagnosis and Treatment of Diabetic Foot Infections. Clinical Infectious Diseases. 2012;54(12):E132-U232.

Figure 1: MIDFUT trial design

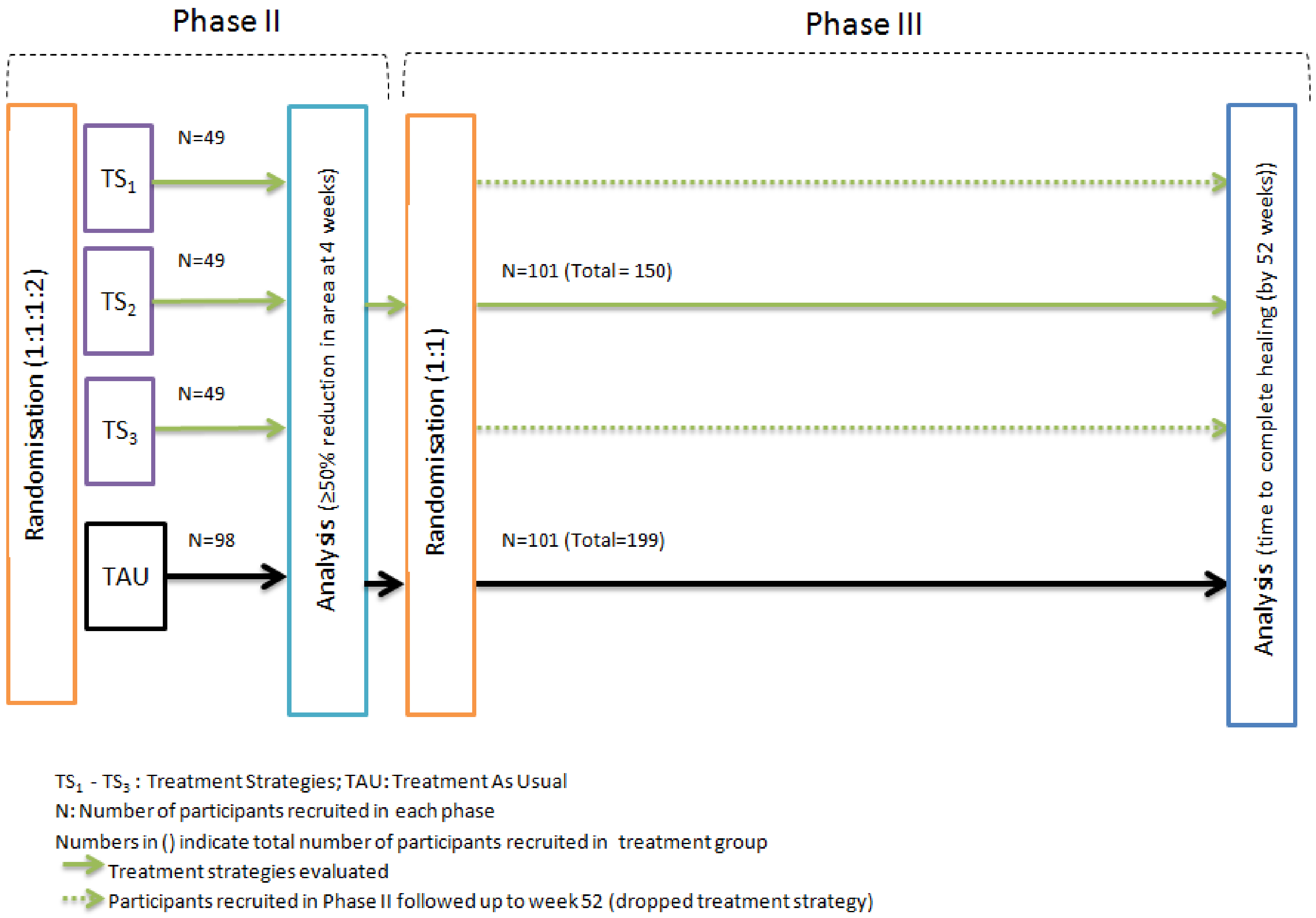

Figure 2: Flow Diagram

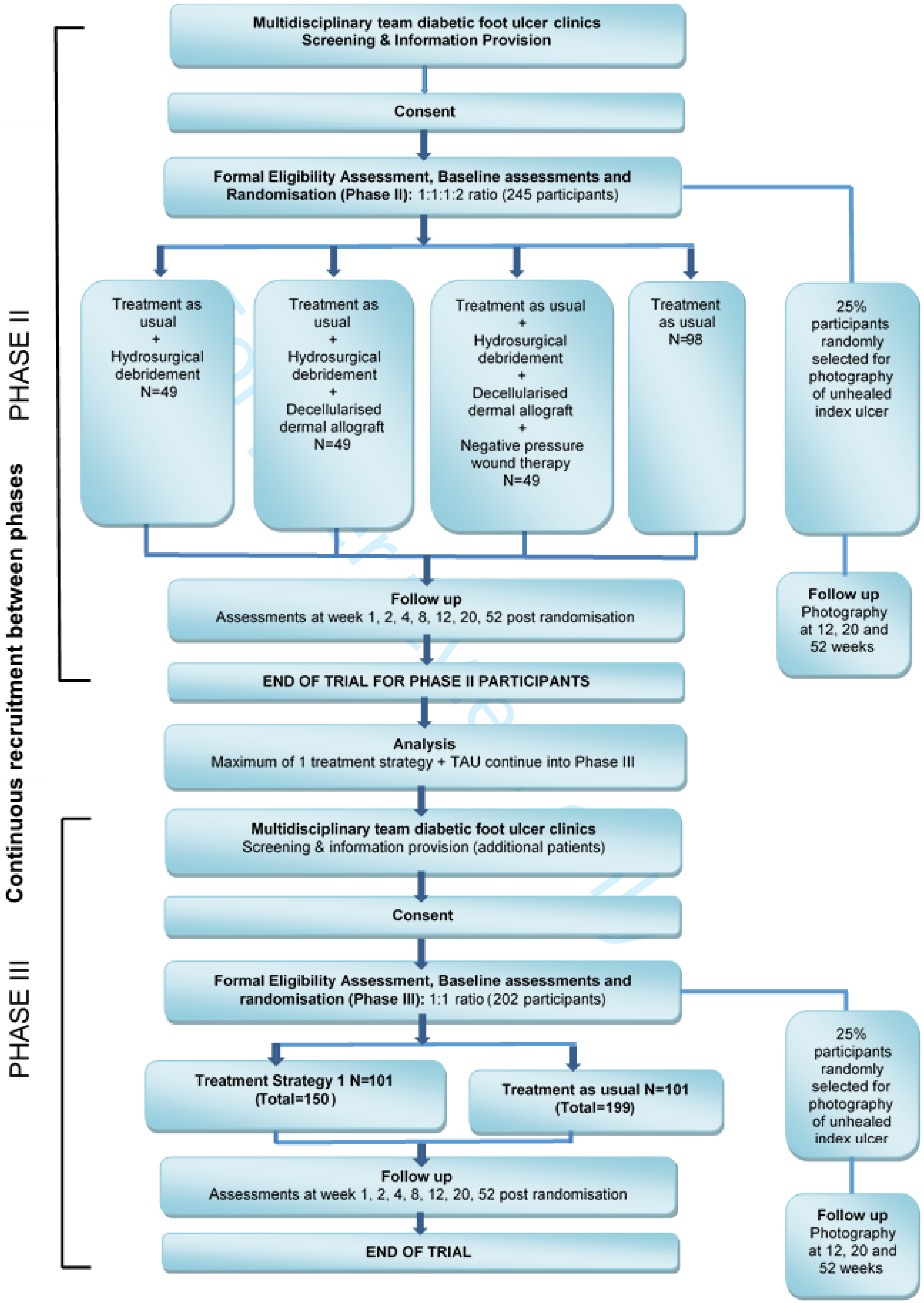

Table 1 Inclusion and Exclusion criteria

Inclusion criteria

1. Aged ≥ 18 years
2. Diagnosis of Diabetes Mellitus (according to WHO criteria (47))
3. Has a chronic DFU or surgical debridement wound or open minor amputation and in the opinion of the attending clinical team is not on a healing trajectory despite usual best care for a minimum of 4 weeks since initial presentation at the MDT DFU service*
4. The index DFU has an area ≥0.8cm^2
5. Ankle brachial index for the leg of the index ulcer ≥0.7 or non-compressible (measurements available in the participants notes taken within 3 months of randomisation can be used if no change in intervention or vascular events have occurred)
6. Expected to comply with the treatment strategies and follow up schedule
7. Consent to foot and wound photography
8. Consent to participate (written/witnessed verbal informed consent)

*Defined as failure to achieve >50% reduction in index ulcer area over a minimum of 4 weeks using local wound measurement policies.

Exclusion criteria

1. Has any current clinically infected DFU on the foot of the index ulcer (as per IDSA guidelines (48))
2. HbA1c>110mmol/mol (measurements available in the participants notes taken within 3 months of randomisation can be used if no change in intervention or vascular events have occurred)
3. Estimated glomerular filtration rate (eGFR) < 20mL/min/1.73m^2 (measurements taken within 3 months of randomisation can be used if no change in intervention or vascular events have occurred)
4. Index ulcer duration >2 years
5. Planned or previous treatment with corticosteroids to an equivalent dose of prednisolone >10mg per day or other immunosuppressive/immunomodulating therapy within 4 weeks prior to randomisation
6. Has evidence of connective tissue disorders as a cause of ulceration (e.g. vasculitis or rheumatoid arthritis)
7. Has evidence of dermatological disorders as a cause of ulceration (e.g. pyoderma gangrenosum or epidermolysis bullosa)
8. Planned or previous growth factor treatment within 4 weeks prior to randomisation
9. Planned or previous revascularisation or foot surgery affecting healing on the foot of the index ulcer within the 4 weeks prior to randomisation
10. Index ulcer base has bone or joint involvement
11. Previously received DCD for the index ulcer within 4 weeks prior to randomisation
12. Previously received NPWT for the index ulcer within 4 weeks prior to randomisation
13. Previously received hydrosurgical or surgical debridement for the index ulcer within 4 weeks prior to randomisation
14. Has previously been **randomised** to the MIDFUT study
15. Unable to receive one or more of the randomised treatment strategies for any reason at the discretion of the attending clinical team (e.g. risk of excessive bleeding, serious falls risk, known allergies to NPWT dressings or dCELL dermis preparation components)

For peer review only

Table 2 Phase II to Phase III Progression Criteria

The progression criteria for the selection of treatment strategies into Phase III is based on the following process:

1) Calculate the point estimate for the proportion of patients with ≥50% ulcer area reduction at 4 weeks post randomisation in all 4 arms (Phase II endpoint). Drop treatment strategy arms for which this proportion is less than 10% higher (on the absolute scale) than that of TAU. That is, if the proportion for TAU is 39% then recommend dropping treatment strategies for which the proportion is less than 49%. Rank the remaining treatment strategies in order of clinical efficacy.

Note: An absolute improvement of 10% in the proportion of patients with ≥50% ulcer area reduction at 4 weeks post randomisation corresponds to the critical cut-point for the selection of treatment strategies based on clinical activity.

2) If more than one treatment strategy has a success rate at 4 weeks of at least 10% greater than TAU then summarise SAEs and rank order of treatment strategies in terms of their safety profile. Only AEs and SAEs that are classified as expected and related to DFUs or trial treatment strategies, or 'related and unexpected SAEs' (RUSAEs) will be considered. Decision on whether to drop treatment strategies with the "least favourable" safety profile will be made by the DMEC.

3) If more than one treatment strategy remains after stages 1) and 2) then summarise treatment-related costs up to 4 weeks and rank order of treatment strategies by ascending cost of treatment versus TAU. Decision on whether to drop the treatment strategies with the highest cost will be made by the DMEC.

4) If no treatment strategies remain, recommend terminating the trial. If one remains, take this forward to Phase III. If more than one treatment strategy satisfies 1) to 3), take forward the top performing arm defined by clinical efficacy (the extent of improvement in the proportion of patients achieving the Phase II endpoint).

The trial will also have a futility rule to allow for stopping of the trial on the basis of no treatment strategy demonstrating at least 10% absolute improvement in the success rate of the Phase II primary outcome. This will be non-binding to allow the DMEC to make the final recommendations to the TSC on whether or not to stop the trial.

For peer review only

CONSORT 2010 checklist of information to include when reporting a randomised trial*

Section/Topic	Item No	Checklist item	Reported on page No
**Title and abstract**			
	1a	Identification as a randomised trial in the title	1
	1b	Structured summary of trial design, methods, results, and conclusions (for specific guidance see CONSORT for abstracts)	1
**Introduction**			
Background and objectives	2a	Scientific background and explanation of rationale	2-3
	2b	Specific objectives or hypotheses	3-4
**Methods**			
Trial design	3a	Description of trial design (such as parallel, factorial) including allocation ratio	4
	3b	Important changes to methods after trial commencement (such as eligibility criteria), with reasons	12
Participants	4a	Eligibility criteria for participants	16
	4b	Settings and locations where the data were collected	5
Interventions	5	The interventions for each group with sufficient details to allow replication, including how and when they were actually administered	6-7
Outcomes	6a	Completely defined pre-specified primary and secondary outcome measures, including how and when they were assessed	9-11
	6b	Any changes to trial outcomes after the trial commenced, with reasons	N/A
Sample size	7a	How sample size was determined	8
	7b	When applicable, explanation of any interim analyses and stopping guidelines	4, 17
Randomisation:			
Sequence generation	8a	Method used to generate the random allocation sequence	5
	8b	Type of randomisation; details of any restriction (such as blocking and block size)	5
Allocation concealment mechanism	9	Mechanism used to implement the random allocation sequence (such as sequentially numbered containers), describing any steps taken to conceal the sequence until interventions were assigned	5
Implementation	10	Who generated the random allocation sequence, who enrolled participants, and who assigned participants to interventions	5
Blinding	11a	If done, who was blinded after assignment to interventions (for example, participants, care providers, those	7-8

For peer review only

		assessing outcomes) and how	
	11b	If relevant, description of the similarity of interventions	N/A
Statistical methods	12a	Statistical methods used to compare groups for primary and secondary outcomes	9-11
	12b	Methods for additional analyses, such as subgroup analyses and adjusted analyses	9-11
**Results**			
Participant flow (a diagram is strongly recommended)	13a	For each group, the numbers of participants who were randomly assigned, received intended treatment, and were analysed for the primary outcome	8, 18, 19
	13b	For each group, losses and exclusions after randomisation, together with reasons	N/A
Recruitment	14a	Dates defining the periods of recruitment and follow-up	13
	14b	Why the trial ended or was stopped	N/A
Baseline data	15	A table showing baseline demographic and clinical characteristics for each group	N/A
Numbers analysed	16	For each group, number of participants (denominator) included in each analysis and whether the analysis was by original assigned groups	N/A
Outcomes and estimation	17a	For each primary and secondary outcome, results for each group, and the estimated effect size and its precision (such as 95% confidence interval)	N/A
	17b	For binary outcomes, presentation of both absolute and relative effect sizes is recommended	N/A
Ancillary analyses	18	Results of any other analyses performed, including subgroup analyses and adjusted analyses, distinguishing pre-specified from exploratory	N/A
Harms	19	All important harms or unintended effects in each group (for specific guidance see CONSORT for harms)	N/A
**Discussion**			
Limitations	20	Trial limitations, addressing sources of potential bias, imprecision, and, if relevant, multiplicity of analyses	1
Generalisability	21	Generalisability (external validity, applicability) of the trial findings	13
Interpretation	22	Interpretation consistent with results, balancing benefits and harms, and considering other relevant evidence	N/A
**Other information**			
Registration	23	Registration number and name of trial registry	1
Protocol	24	Where the full trial protocol can be accessed, if available	13
Funding	25	Sources of funding and other support (such as supply of drugs), role of funders	14

*We strongly recommend reading this statement in conjunction with the CONSORT 2010 Explanation and Elaboration for important clarifications on all the items. If relevant, we also recommend reading CONSORT extensions for cluster randomised trials, non-inferiority and equivalence trials, non-pharmacological treatments, herbal interventions, and pragmatic trials. Additional extensions are forthcoming: for those and for up to date references relevant to this checklist, see www.consort-statement.org.

For peer review only

BMJ Open

Multiple Interventions for Diabetic Foot Ulcer Treatment Trial (MIDFUT): Study Protocol for a randomised controlled trial

Journal:	*BMJ Open*
Manuscript ID	bmjopen-2019-035947.R1
Article Type:	Protocol
Date Submitted by the Author:	11-Dec-2019
Complete List of Authors:	Brown, Sarah; University of Leeds, Clinical Trials Research Unit<br>Nixon, Jane; University of Leeds, Clinical Trials Research Unit<br>Ransom, Myka; University of Leeds, Clinical Trials Research Unit<br>Gilberts, Rachael; University of Leeds, Clinical Trials Research Unit<br>Dewhirst, Nikki; Leeds Teaching Hospitals NHS Trust, Leeds Vascular Institute; University of Leeds, Clinical Trials Research Unit<br>McGinnis, Elizabeth; Leeds Teaching Hospitals NHS Trust<br>Longo, Roberto; University of Leeds, Academic Unit of Health Economics<br>Game, Frances; National Health Service, Department of Diabetes<br>Bojke, Chris; University of Leeds, Academic Unit of Health Economics<br>Chadwick, Paul; College of Podiatry<br>Chandrasekar, Akila; NHS Blood and Transplant, Tissue Services<br>Chetter, IC; University of Hull<br>Collier, Howard; University of Leeds, Clinical Trials Research Unit<br>Fernandez, Catherine; University of Leeds Faculty of Medicine and Health, Clinical Trials Research Unit<br>Homer-Vanniasinkam, Shervanthi; Leeds Teaching Hospitals NHS Trust<br>Jude, Edward; Tameside General Hospital, Diabetes Centre<br>Leigh, Richard; Royal Free London NHS Foundation Trust<br>Lomas, Richard; NHS Blood and Transplant, Tissue Services<br>Vowden, Peter; Bradford Teaching Hospitals NHS Foundation Trust,<br>Wason, James; Medical Research Council, Biostatistics Unit<br>Sharples, Linda; London School of Hygiene and Tropical Medicine, Medical Statistics<br>Russell, David; Leeds Teaching Hospitals NHS Trust, Leeds Vascular Institute; University of Leeds Leeds Institute of Genetics Health and Therapeutics,
**Primary Subject Heading**:	Diabetes and endocrinology
Secondary Subject Heading:	Research methods
Keywords:	Diabetic foot < DIABETES & ENDOCRINOLOGY, DIABETES & ENDOCRINOLOGY, WOUND MANAGEMENT, STATISTICS & RESEARCH METHODS, Clinical trials < THERAPEUTICS

SCHOLARONE™
Manuscripts

Multiple Interventions for Diabetic Foot Ulcer Treatment Trial (MIDFUT): Study Protocol for a randomised controlled trial

Brown, Sarah[1]; Nixon, Jane[1]; Ransom, Myka[1]; Gilberts, Rachael[1]; Dewhirst, Nikki[1,2]; McGinnis, Elizabeth[1,2]; Longo, Roberto[1]; Game, Frances[3]; Bojke, Chris[1]; Chadwick, Paul[4]; Chandrasekar, Akila[5]; Chetter, Ian[6]; Collier, Howard[1]; Fernandez, Catherine[1]; Homer-Vanniasinkam, Shervanthi[2]; Jude, Edward[7]; Leigh, Richard[8]; Lomas, Richard[5]; Vowden, Peter[9]; Wason, James[10,12]; Sharples, Linda[11]; Russell, David[1,2];

1Clinical Trials Research Unit, Leeds Institute of Clinical Trials Research, University of Leeds, UK; [2]Leeds Teaching Hospitals NHS Trust, UK; [3]Derby Teaching Hospitals NHS Foundation Trust, UK; [4]College of Podiatry, London, UK; [5]NHS Blood and Transplant, Liverpool, UK; [6]University of Hull, UK; [7]Tameside General Hospital, Manchester, UK; [8]Royal Free London NHS Foundation Trust, UK; [9]Bradford Teaching Hospitals NHS Foundation Trust, UK; [10]University of Cambridge, UK; [11]London School of Hygiene and Tropical Medicine, UK; [12] University of Newcastle, UK.

Corresponding author:
Miss Sarah Brown
Clinical Trials Research Unit
University of Leeds
Leeds
LS29JT
Email: medsbro@leeds.ac.uk
Tel: 0113 343 1475
Fax: 0113 343 1471

Keywords: Diabetic Foot, Diabetes & Endocrinology, Wound Management, Statistics & Research Methods, Clinical Trials

Word count: 7,762

Abstract
Introduction: Diabetes affects more than 425 million people worldwide with a lifetime risk of diabetic foot ulcer (DFU) of up to 25%. Management includes wound debridement, wound dressings, off-loading, treatment of infection and ischaemia, optimising glycaemic control; use of advanced adjuvant therapies is limited by high cost and lack of robust evidence.

Methods and Analysis: A multicentre, seamless Phase II/III, open, parallel group, multi-arm multi-stage randomised controlled trial in patients with a hard-to-heal DFU, with blinded outcome assessment. A maximum of 447 participants will be randomised (245 participants in Phase II and 202 participants in Phase III).

The Phase II primary objective will determine the efficacy of treatment strategies including hydrosurgical debridement +/- decellularised dermal allograft, or the combination with negative pressure wound therapy, as an adjunct to treatment as usual (TAU), compared to TAU alone, with patients randomised in a 1:1:1:2 allocation. The outcome is achieving at least 50% reduction in index ulcer area at 4 weeks post randomisation.

The Phase III primary objective will determine whether one treatment strategy, continued from Phase II, reduces time to healing of the index ulcer compared with TAU alone, with participants randomised in a 1:1 allocation. Secondary objectives will compare healing status of the index ulcer, infection rate, re-ulceration, quality of life, cost-effectiveness and incidence of adverse events over 52 weeks post randomisation.

For peer review only

Phase II and III primary endpoint analysis will be conducted using a mixed-effects logistic regression model and Cox Proportional Hazards regression respectively.

A within-trial economic evaluation will be undertaken; the primary economic analysis will be a cost-utility analysis presenting ICERs for each treatment strategy in rank order of effectiveness, with effects expressed as QALYs.

The trial has pre-defined progression criteria for the selection of one treatment strategy into Phase III based on efficacy, safety and costs at 4 weeks.

Ethics and Dissemination: Ethics approval has been granted by NRES Committee Yorkshire & The Humber - Bradford Leeds Research Ethics Committee; approved 26th April 2017; (REC reference: 17/YH/0055). There is planned publication of a monograph in NIHR journals and main trial results and associated papers in high-impact peer reviewed journals.

Trial registration: ISRCTN64926597; Registered on 06/06/2017.

Article Summary
Strengths and Limitations of this study:

- The multi-arm multi-stage design will allow early evaluation of multiple treatment strategies in a Phase II/III design, stopping treatments which fail to demonstrate sufficient improvement, evaluating only those showing greatest efficacy in a Phase III trial;
- Comparison of multiple treatment strategies to a shared control group, thus requiring fewer participants compared to conventional trial designs;
- Clear pre-defined progression criteria for the selection of the treatment strategy into Phase III;
- Pragmatic in the identification of patients with hard to heal ulcers;
- The target sample size allows only one treatment strategy to be taken forward into Phase III.

For peer review only

Introduction

Diabetes currently affects more than 425 million people worldwide (1), and this is expected to increase to 629 million by 2045 (2). A total of 21-30% of patients with diabetes develop peripheral neuropathy or lose sensation in their feet (3, 4) and extrapolation from incidence studies suggests that lifetime incidence of diabetic foot ulcer (DFU) may be as high as 25% (5). More than 50% of DFUs become infected, requiring hospitalisation, and 20% of infections result in amputation (6), contributing approximately 80% of non-traumatic amputations performed in the developed world (7).

In the UK, diabetes affects 4.5 million people (8) with approximately 64,000 having a DFU at any one time (9). In 2014-15 NHS England spent an estimated £1 billion on DFU treatment (10). This does not take into account the costs imposed on the public sector and society as a whole through working days lost, reductions in tax revenue, increases in benefit payments and social care resources. Furthermore, DFUs have a major impact upon patient health related quality of life (HRQoL), including impaired physical function, mental wellbeing and social interaction (11) .

Management of DFUs comprises provision of NICE recommended best 'treatment as usual' (TAU) through multi-disciplinary team (MDT) DFU clinics (podiatrists, diabetologists, vascular surgeons etc.) and concomitant treatment strategies including: optimising glycaemic control, sharp non-surgical debridement, dressing application, off-loading, treatment of infection and ischaemia (12). There are a number of advanced/adjuvant therapies but their use is limited by high unit cost and an absence of robust evidence.

Despite implementation of MDT care, a national audit of over 33,000 ulcers reports 48.3% remain unhealed at 12 weeks (13). Of those patients with less than the median reduction in DFU area at 4 weeks (<53% reduction), only 9% went onto heal at 12 weeks (14), whilst those in the top half for healing at 4 weeks (≥53% reduction) had a higher probability of healing of 58% at 12 weeks. Delayed healing increases the probability of adverse sequelae including infection and amputation(6). Drivers of the cost of care change with the need for hospitalisation: in a recent study community nurse visits accounted for 65% of total costs for healed and unhealed wounds managed in the outpatient setting, whereas 65% of costs in patients having amputation were incurred in secondary care (15). Thus implementation of adjuvant therapies, which are often more costly, is more likely to be cost effective in those patients identified as "hard to heal" (failing to decrease by >50% at 4 weeks), whereas those DFUs reaching >50% healing at 4 weeks are likely to heal without the need for more expensive interventions. Those DFU's reported as unhealed at 12 weeks in the 2019 UK National Diabetic Foot Audit (13) may have benefitted from such therapies.

Establishing efficacious adjuvant therapies for use in non-healing wounds is a priority to improve healing rates and HRQoL, and reduce the risk of morbidity and cost. There is a paucity of high quality trials assessing adjuvant wound therapies in DFUs and NICE guideline NG19 and others have highlighted the need for randomised controlled trials (RCTs) of negative pressure wound therapy (NPWT) and other adjuvant therapies (12, 16). Technological advances in three adjuvant therapies mean they are now available for routine clinic use.

1) NPWT is available in a small portable pump which doesn't restrict patient movement.
2) Sufficient surgical debridement which changes a chronic wound biology to an acute wound can now be undertaken using hydrosurgical debridement (HD) under local anaesthetic in clinic, enhancing patient experience and reducing costs by avoiding additional hospital visits for day-case surgery. This also allows wound bed preparation to a "graft ready state" for advanced wound adjuncts, a state which cannot be achieved by less aggressive debridement with wound debridement pads such as Debrisoft®. HD has been shown to be as effective as operating theatre surgical debridement in wound healing outcomes (17).
3) Decellularised dermal allograft (DCD) has been used in the USA for treatment of DFU, with improved healing compared to standard care (18, 19) but cost has been prohibitive in the UK.

For peer review only

A novel DCD, prepared from skin donated by voluntary UK deceased donors has recently been developed within the NHS, is approved by the Human Tissue Authority (HTA) and available for use in the UK. DCD is prepared and supplied by NHS Blood and Transplant (a Department of Health Special Health Authority). However, the application of DCD requires surgical debridement to a 'graft ready wound bed' and it is not known whether the surgical debridement alone leads to improved healing in this setting.

Performing multiple RCTs to assess each intervention individually would be time consuming and expensive. Moreover, these therapies are often used in combination. The MIDFUT trial utilises an efficient, informative and ethical, adaptive, multi-arm, multi-stage (MAMS) design (20). This involves early evaluation of combinations of the candidate interventions in a Phase II/III design, stopping recruitment to treatment strategies which fail to demonstrate sufficient improvements in DFU healing, using an intermediate endpoint at 4 weeks post randomisation, and evaluating only one treatment strategy showing greatest efficacy in a Phase III trial.

The evidence for adjuvant therapies for DFU treatment was reviewed in NICE guideline NG19 (12), which concluded that the quality of trials was poor, due to binary and early endpoints and small sample sizes. It recommended that future trials are sufficiently powered, with outcomes including time to healing, incidence and extent of amputation (major or minor), ulcer recurrence, HRQOL, adverse events, hospital admissions and length of stay. These findings were supported by a review by the International Working Group on the Diabetic Foot (21).

In summary, quality data on the outcome of adjuvant therapies for DFU is rare. A 30% increase in prevalence of disease is anticipated by 2035, which will add to the already substantial costs of treating DFUs, in a time of global fiscal uncertainty. With international guidance advocating the need for robust RCTs in this area (21), and technical advancements in three adjuvant therapy options (HD, DCD and NPWT), an RCT to compare treatment strategies is timely.

Objectives

In Phase II, the aim is to identify the most promising of the three treatment strategies compared to treatment as usual (TAU) using short-term efficacy and in Phase III, to investigate the clinical and cost effectiveness of one treatment strategy from Phase II compared to TAU in the treatment of patients with hard to heal DFUs.

Phase II Primary Objective

To determine the efficacy of the treatment strategies,

1) TAU + HD alone
2) TAU + HD + DCD
3) TAU + HD + NWPT + DCD

compared to TAU alone using the short-term intermediate outcome of achieving at least 50% reduction in index ulcer area at 4 weeks post randomisation (Phase II primary endpoint).

Phase III Primary Objective

To determine whether one treatment strategy continued from Phase II, as an adjunct to TAU reduces time to healing of the index ulcer compared with TAU alone.

The primary endpoint is time *to healing of the index ulcer* from randomisation to the date the index ulcer is confirmed as healed at the first confirmation visit conducted by the blinded assessor (providing the index ulcer is confirmed as healed at a second clinical assessment two weeks later).

For peer review only

Phase III Secondary Objectives:
To compare one treatment strategy as an adjunct to TAU, continued from Phase II, with TAU alone for:

- healing status of the index ulcer at 12, 20 and 52 weeks
- rate of ulcer infection in the foot of the index ulcer over 52 weeks post randomisation
- incidence of re-ulceration following healing of index ulcer over 52 weeks post randomisation
- quality of life using Diabetic Foot Ulcer Scale Short Form (DFS-SF) and EuroQol - five dimensions (EQ-5D-5L) over 52 weeks post randomisation
- incidence of adverse events (including amputation, infection in any ulcer on the foot of the index ulcer and hospital admission) over 52 weeks post randomisation
- cost effectiveness over 52 weeks

Phase III Exploratory Objective:
To explore factors prognostic of ulcer healing

Methods

The MIDFUT trial is a multi-centre, seamless Phase II/III, open, parallel group, multi-arm multi-stage (MAMS) randomised controlled trial (RCT) in patients with a hard to heal DFU, with blinded outcome assessment.

The MAMS trial design will allow an early evaluation of three candidate treatment strategies in a Phase II/III design. Randomisation to treatment strategies that fail to demonstrate sufficient improvement in index ulcer healing at the end of Phase II will be stopped. Only one treatment strategy showing greatest early efficacy will undergo clinical and cost-effectiveness assessment in Phase III (see Figure 1 for a schematic of the design).

Three treatment strategies will be compared to TAU in Phase II. Treatment strategies for which the estimated proportion of responders is less than 10% higher than the proportion of responders on TAU (absolute difference) will be dropped at the end of Phase II (response defined as achieving at least 50% reduction in wound area of the index ulcer). One treatment strategy and TAU will be evaluated in Phase III. If more than one treatment strategy shows a sufficient response in Phase II, then the decision on which treatment strategy to evaluate in Phase III will consider information on the safety profile and costs of the treatment strategies up to 4 weeks post randomisation.

A maximum of 447 participants will be recruited, 245 participants in Phase II and at most 220 participants in Phase III. Recruitment at centres will continue without interruption between Phase II and Phase III.

All participants from Phases II and III will be followed up at weeks 1, 2, 4, 8, 12, 20 and 52 post-randomisation (including those where healing of the index ulcer has been confirmed), or week 54 where healing of the index ulcer is first reported at week 52 (see Figure 2).

The trial includes a 9-month internal pilot phase in Phase II to evaluate the feasibility of recruitment and therefore delivery of the trial.

Moreover, a Study Within a Trial (SWaT) will be included to determine the extent of agreement in the assessment of healing between central blinded photography review and the clinical assessment of healing.

An interim analysis will be conducted after 220 patients have reached 52 weeks post-randomisation to re-estimate the overall loss to follow-up rate and the final sample size. The review will be conducted in a blinded manner.

For peer review only

Recruitment/Consent

The trial will be conducted in secondary care and community clinics that provide a MDT-DFU service (which includes as a minimum a clinician trained in each trial intervention, podiatrist, diabetologist, vascular surgeon and orthotist).

Patients under the care of the MDT-DFU out-patient clinics, with a current DFU, surgical debridement wound or minor amputation wound, will be assessed for eligibility in accordance with the criteria in Table 1.

Table 1 Inclusion and Exclusion criteria

Inclusion criteria

1. Aged ≥ 18 years
2. Diagnosis of Diabetes Mellitus (according to WHO criteria (22))
3. Has a chronic DFU or surgical debridement wound or open minor amputation and in the opinion of the attending clinical team is not on a healing trajectory despite usual best care for a minimum of 4 weeks since initial presentation at the MDT DFU service*
4. The index DFU has an area ≥0.8cm^2
5. Ankle brachial index for the leg of the index ulcer ≥0.7 or non-compressible (measurements available in the participants notes taken within 3 months of randomisation can be used if no change in intervention or vascular events have occurred)
6. Expected to comply with the treatment strategies and follow up schedule
7. Consent to foot and wound photography
8. Consent to participate (written/witnessed verbal informed consent)

*Defined as failure to achieve >50% reduction in index ulcer area over a minimum of 4 weeks using local wound measurement policies.

Exclusion criteria

1. Has any current clinically infected DFU on the foot of the index ulcer (as per IDSA guidelines (23))
2. HbA1c>110mmol/mol (measurements available in the participants notes taken within 3 months of randomisation can be used if no change in intervention or vascular events have occurred)
3. Estimated glomerular filtration rate (eGFR) < 20mL/min/1.73m^2 (measurements taken within 3 months of randomisation can be used if no change in intervention or vascular events have occurred)
4. Index ulcer duration >2 years
5. Planned or previous treatment with corticosteroids to an equivalent dose of prednisolone >10mg per day or other immunosuppressive/immunomodulating therapy within 4 weeks prior to randomisation
6. Has evidence of connective tissue disorders as a cause of ulceration (e.g. vasculitis or rheumatoid arthritis)
7. Has evidence of dermatological disorders as a cause of ulceration (e.g. pyoderma gangrenosum or epidermolysis bullosa)
8. Planned or previous growth factor treatment within 4 weeks prior to randomisation
9. Planned or previous revascularisation or foot surgery affecting healing on the foot of the index ulcer within the 4 weeks prior to randomisation
10. Index ulcer base has bone or joint involvement
11. Previously received DCD for the index ulcer within 4 weeks prior to randomisation
12. Previously received NPWT for the index ulcer within 4 weeks prior to randomisation
13. Previously received hydrosurgical or surgical debridement for the index ulcer within 4 weeks prior to randomisation
14. Has previously been **randomised** to the MIDFUT study

For peer review only

15. Unable to receive one or more of the randomised treatment strategies for any reason at the discretion of the attending clinical team (e.g. risk of excessive bleeding, serious falls risk, known allergies to NPWT dressings or dCELL dermis preparation components)

Potentially eligible patients will receive a verbal explanation of the study and a Patient Information Leaflet (PIL) by the attending clinical/research team. Strategies to encourage recruitment include:

- posters and/or leaflets in clinic waiting areas and other appropriate locations,
- letter and PIL sent to patients with their out-patient appointment letter,
- study included on relevant websites and research databases that can be accessed by members of the public,
- ethically approved tweets on Twitter.

Following information provision, patients will have as long as they need to consider participation and to discuss the study with their family and other healthcare professionals before consent to participate in the study is requested.

Assenting patients will be invited to provide informed consent and complete an eligibility assessment. Full informed consent will be obtained for all participants prior to the participant undergoing procedures that are specifically for the purposes of the study and are not part of TAU at the participating centre.

Witnessed consent by a representative who is independent of the trial will be available where relevant.

Patients who provide written/witnessed verbal informed consent, but subsequently lose capacity will be withdrawn from the trial.

Non-randomised patients
Participating research sites will complete a log of all patients presenting with a DFU and considered for the trial, but not recruited. Anonymised information to be collected includes age, sex, ethnicity, reason not eligible or reason declined participation.

Randomisation
Following confirmation of eligibility, consent and completion of baseline assessments, participants will be randomised. In Phase II, randomisation will be in a 1:1:1:2 allocation ratio to the three treatment strategies and TAU group respectively, as an approximation to Dunnett's recommendation (24). In Phase III, randomisation will be in a 1:1 allocation ratio to one treatment strategy and TAU. Randomisation in both phases will use a minimisation algorithm, incorporating a random element, via a central 24-hour automated telephone or internet randomisation system, based at the Leeds Clinical Trials Research Unit (CTRU). The dynamic allocation method will ensure the groups are well balanced for:

- Centre
- Aetiology (neuropathic or neuro-ischaemic)
- Index ulcer duration (<6 months, ≥ 6 months)
- Anatomical site (forefoot, mid/hindfoot)
- Presentation (DFU, surgical debridement wound, open minor amputation)

In addition, at the time of randomisation, 25% of participants will be randomly selected to have photographs of the index ulcer taken, if it is unhealed, at weeks 12, 20 and 52, for central blinded review.

For peer review only

Blinding
Due to the nature of the treatment strategies it is not possible to blind participants, the clinicians or research team to the treatment group allocation. However, primary outcome assessments will be conducted by an independent clinical assessor with no knowledge of treatment allocation. To mitigate risk of assessment bias the blinded assessor will also have no access to participant notes or trial Case Report Forms (CRFs). Blinding will be maintained when tracings and photographs at week 4, and confirmation of the index ulcer healing assessments, are returned to the CTRU (e.g. through separate mail or independent clerical staff).

For the Phase II primary outcome and Phase III exploratory objective, a blinded assessor at each site will complete an acetate tracing and take a 2D digital photograph of the index ulcer at week 4. Measurements will be obtained from the index ulcer tracing using 'Image J' software (25) by a member of the CTRU team who is independent of the research teams at recruiting sites and blind to treatment allocation. A photograph of the index ulcer will be taken as a back-up in the event that a tracing cannot be taken or is of insufficient quality to determine the index ulcer outline.

For the Phase III primary endpoint, all participants recruited to both Phase II and III will also have a photograph taken of the reported healed index ulcer by the blinded assessor within 3 days of healing being reported and two weeks later as a confirmation of healing, which will undergo blinded central review.

Photographs taken of healed and of unhealed index ulcers for randomly selected participants will be submitted for central blinded photography review by clinical members of the Trial Management Group who will not be aware of the participant's identity, treatment group or time point at which the photograph was taken.

Interventions
All randomised treatment strategies will be applied to the index ulcer as a "once only intervention" on the day of randomisation in the MDT-DFU service clinic, with the exception of NPWT which will be applied until the 2-week visit. Treatment of any other ulcers will continue as per the treating clinician decision.

At baseline, randomisation and each follow-up visit all participants will receive TAU. At the randomisation treatment visit, the participant will be randomised to receive the treatment strategy specific to the arm of the trial for the index ulcer. This will include one or more of the following:

Treatment as usual (TAU)
Participants will receive the minimum standard care provided by the recruiting centre. This will be in line with NICE guidelines (12) and is likely to include attendance at the MDT-DFU service clinic(s) at least fortnightly until healing is confirmed for wound assessment, sharp non-surgical debridement of callous/non-viable tissue, review of off-loading and to optimise diabetes and wound assessment as required, including community services visits, typically 1 to 2 times weekly. In line with NICE guidelines, use of removable below-knee walking device or removable cast walker will be encouraged. Wound dressing changes will be performed between clinic visits according to local policies.

Hydrosurgical Debridement (HD)
Hydrosurgical Debridement (HD), a one-off procedure on the day of randomisation, applies saline at high pressure via a pump through a hand piece. This has an operating window located at the instrument's distal tip. During operation the flow of pressurised saline creates a local vacuum. As the operating window of the handset is passed over the tissue, non-viable material and debris are removed. The ulcer bed is debrided to healthy bleeding tissue which may require local anaesthetic.

For peer review only

Negative Pressure Wound Therapy (NPWT)

Negative Pressure Wound Therapy (NPWT), applied on the day of randomisation, consists of a foam dressing cut to shape and applied to the wound. An air-tight seal is established with a film dressing; this is then connected to a pump which applies gentle suction to the wound. This allows the removal of fluid from the wound, which is collected in a canister attached to the pump, which is carried by the participant at all times in the bag provided. Alternatively, a self-contained system consisting of a disposable NPWT pump attached to an absorbent adhesive dressing may be used. The dressing is usually changed at least once a week, and the NPWT will be applied for 2 weeks post randomisation.

Decellularised Dermal Allograft (DCD)

Decellularised Dermal Allografts are prepared from split skin grafts obtained from deceased human tissue donors, which are processed and sterilised. Processing retains the normal skin structure, but removes donor cells and cell remnants meaning the graft is not rejected and functions as a permanent tissue replacement. Upon receipt at participating sites, the graft will be stored between 0-40°C until the expiry date stated on the graft label. Prior to application the graft is soaked in a bowl of sterile saline solution for 15 minutes. The graft is then cut to size using sterile scissors and applied directly to the debrided wound bed, epidermal side upwards. Following application, the ulcer is covered with a non-adherent contact layer and a secondary bolster dressing or NPWT (as per randomisation). In those DFUs allocated to DCD, the wound bed is not debrided for 4 months post-treatment, unless clinically indicated, although debridement of wound edge and surrounding tissue can continue as per TAU.

Assessments/data collection and follow-up

Baseline Assessment

Participant demographics including date of birth, gender, ethnicity, NHS number, and site of the index ulcer will be recorded.

Clinical history will be recorded including smoking history, duration and type of diabetes, number of ulcers and index ulcer characteristics e.g. first or recurrent ulcer, aetiology, existing wound therapies and SINBAD classification (26). Initial index ulcer area tracing (using acetate) will also be obtained. Participants will be asked to complete quality of life questionnaires: Diabetic Foot Scale –Short Form (DFS-SF) and the EuroQoL-5D 5 level (EQ-5D-5L).

Randomisation and application of the treatment strategy will take place after baseline assessments and questionnaires have been completed, on the same day.

Information collected post treatment will include details of the treatment strategy applied to the index ulcer and to any other ulcers on the foot of the index ulcer, and expected adverse events (AEs) and serious adverse events (SAEs). Post HD debridement index ulcer area acetate tracing and photographs will be obtained.

Follow-up Assessments

At a routine clinic assessment at week 1 and at week 2, 4, 12, 20 and 52 post-randomisation the following assessments will be conducted by a member of the clinical research team (clinician, clinical research nurse or registered healthcare professional): healing status of the index ulcer, episodes of infection in the foot of the index ulcer (IDSA criteria), revascularisation of the index limb, index ulcer treatments and expected AEs or SAEs. In addition, at weeks 1 and 2 post-randomisation, an assessment of compliance with NPWT and DCD (where applicable), and at weeks 2 to 52 post-randomisation an assessment of re-ulceration of the index ulcer will be conducted. At week 2 and week 4 post randomisation, an acetate tracing and photograph of the index ulcer post sharp non-surgical debridement (where clinically indicated) will be taken; at week 4 this is conducted by a blinded assessor (clinician, research nurse or registered healthcare professional).

For peer review only

Patient questionnaires (DFS-SF, EQ-5D-5L and Health Resource Utilisation (HRU)) will be completed at weeks 4, 12, 20 and 52 post-randomisation; the HRU questionnaire will also be completed at week 8 post-randomisation.

Healing and Re-ulceration Assessments

Healing is defined as complete closure of the ulcer: 100% re-epithelialisation of the wound surface with the absence of drainage, confirmed by blinded assessment of index ulcer healing status at two consecutive assessments two weeks apart (27).

Healing of the index ulcer will be reported in one of the following scenarios;

- By the Research Nurse/Registered Healthcare Professional at a research visit.
- During the participant's routine appointment at the MDT-DFU service clinic, podiatry clinic, GP practice nurse and/or at home by district nurses as per treatment as usual
- Patient self-reporting to the research team or to the attending clinical team in between routine appointments who will then inform the research team.

The attending clinical team will contact the research team to report the date the index ulcer was first noted as healed, who will then arrange an initial visit within 3 days of healing of the index ulcer first being reported and a 2 week follow-up visit (+/- 3 days) with the blinded assessor to assess index ulcer healing status and conduct photography.

Re-ulceration is defined as recurrence of a full thickness break in the epithelium at the same location as the index ulcer (28). Re-ulceration of the index ulcer will be established either by participant self-referral to the research team, at a routine clinic or research appointment or by continuous screening of new referrals to the MDT-DFU service clinic where participants will be flagged to the research team by the attending clinical team. Re-ulceration of the index ulcer will be confirmed by a blinded assessor, within 7 days of re-ulceration being reported, with reference to the photograph of the foot taken at the randomisation visit, photography undertaken and the date of re-ulceration of the index ulcer recorded.

Sample size

The planned maximum sample size is 447 patients, 245 patients in Phase II and 202 patients in Phase III. The apportionment of participants to Phase II and Phase III was estimated using a series of simulation studies.

In Phase II, 49 patients per treatment strategy arm and 98 patients in the TAU arm will be recruited. The target effect size in Phase II is an absolute increase of 25% in the proportion of patients achieving at least a 50% reduction in wound area by 4 weeks post randomisation, assuming 39% reach at least a 50% reduction by week 4 in the TAU arm (local audit data) and 64% achieve this outcome in the treatment strategy arms.

An additional 101 patients will be recruited into each arm evaluated in Phase III, corresponding to a total (Phase II and III combined) of 150 in the remaining treatment strategy group and 199 in the TAU arm (total of 349 patients for evaluation in Phase III).

The minimum clinically important effect size in Phase III is a hazard ratio of 1.5, assuming a median time to healing of 21 weeks for the TAU arm (local audit data) and 14 weeks for the treatment strategy arms (18, 29-32) and 18.0% and 7.6% ***un***healed at 52 weeks in the TAU and treatment strategy group respectively (assuming exponential distribution for time to healing).

Several scenarios for the power of the trial have been considered. In all cases a 10% loss to follow-up by 4 weeks and 25% loss to follow up by 52 weeks is assumed. In the case where there is a single effective treatment strategy arm, the design has 83% power to recommend a truly effective treatment strategy (i.e. for it to progress from Phase II and for a significant result found at Phase III). A

For peer review only

treatment strategy group that progresses to Phase III and which is significantly better than TAU at the 2-sided 4% significance level (to control the family wise error rate at 5%) on the time to healing endpoint will be declared clinically effective.

A formal sample size review will be conducted at 52 weeks, after 220 patients have been recruited, to re-estimate the proportion of patients lost to follow-up by 52 weeks post randomisation and the final sample size. The review will allow the overall loss to follow-up to be estimated to a minimum precision ±5.7% (corresponding to half width of the 95%CI), assuming a maximum loss to follow-up of 25%.

Progression criteria for Phase III

The minimum criterion for taking treatment strategies forward into Phase III will be defined as at least a 10% increase in the probability of achieving ≥50% reduction in index ulcer area at 4 weeks post randomisation above that observed for TAU, corresponding to the minimum clinically important difference (clinical opinion). If more than one treatment strategy passes this threshold at Phase II then the selection criteria will be based on a combination of efficacy, safety profile and cost of treatment strategies up to 4 weeks post-randomisation. The progression criteria are provided in further detail in Table 2.

Table 2 Phase II to Phase II Progression Criteria

The progression criteria for the selection of treatment strategies into Phase III is based on the following process:

1) Calculate the point estimate for the proportion of patients with ≥50% ulcer area reduction at 4 weeks post randomisation in all 4 arms (Phase II endpoint). Drop treatment strategy arms for which this proportion is less than 10% higher (on the absolute scale) than that of TAU. That is, if the proportion for TAU is 39% then recommend dropping treatment strategies for which the proportion is less than 49%. Rank the remaining treatment strategies in order of clinical efficacy.

Note: An absolute improvement of 10% in the proportion of patients with ≥50% ulcer area reduction at 4 weeks post randomisation corresponds to the critical cut-point for the selection of treatment strategies based on clinical activity.

2) If more than one treatment strategy has a success rate at 4 weeks of at least 10% greater than TAU then summarise SAEs and rank order of treatment strategies in terms of their safety profile. Only AEs and SAEs that are classified as expected and related to DFUs or trial treatment strategies, or 'related and unexpected SAEs' (RUSAEs) will be considered. Decision on whether to drop treatment strategies with the "least favourable" safety profile will be made by the Data Monitoring and Ethics Committee (DMEC).

3) If more than one treatment strategy remains after stages 1) and 2) then summarise treatment-related costs up to 4 weeks and rank order of treatment strategies by ascending cost of treatment versus TAU. Decision on whether to drop the treatment strategies with the highest cost will be made by the DMEC.

4) If no treatment strategies remain, recommend terminating the trial. If one remains, take this forward to Phase III. If more than one treatment strategy satisfies 1) to 3), take forward the top performing arm defined by clinical efficacy (the extent of improvement in the proportion of patients achieving the Phase II endpoint).

The trial will also have a futility rule to allow for stopping of the trial on the basis of no treatment strategy demonstrating at least 10% absolute improvement in the success rate of the Phase II primary outcome. This will be non-binding to allow the DMEC to make the final recommendations to the TSC on whether or not to stop the trial.

Statistical analysis

For peer review only

Statistical analysis plans for Phase II and Phase III final analyses will be finalised and signed off before any data analyses are conducted.

The complete case population will be used for the analysis of the Phase II endpoint under the assumption that data are missing at random (MAR) (33). A sensitivity analysis will be considered if there is differential missing endpoint data observed across treatment arms.

Phase III analyses will use Intention to Treat (ITT) whereby patients will be analysed according to randomised treatment group. A per-protocol population will also be defined.

For Phase III endpoint analyses, data from all participants recruited in Phase II and Phase III will be included.

Phase II Primary Endpoint Analysis

Primary analysis

Treatment effects and 95% CI on response at 4 weeks will be estimated from multivariable mixed-effects logistic regression, including the minimisation factors and treatment group as fixed effects and centres as random effects. Simple contrasts for each treatment strategy compared to the TAU arm will be used.

Phase II secondary endpoint analysis

AEs and SAEs that are classified as expected and related to DFUs and trial treatment strategies, or 'related and unexpected SAEs' (RUSAEs) will be summarised by treatment group.

Mean per-patient costs of treatment, health care use and total resource use, together with a measure of variance will be reported by treatment group.

Phase III Primary Endpoint Analysis

Primary analysis

The hazard ratios for the Phase III endpoint will be estimated using Cox Proportional Hazards regression with covariates for the minimisation factors, treatment arm (fixed effects) and centre (random effects) and stratification for the phase in which the patient was recruited.

Phase III secondary endpoint analysis

Similar regression-based analyses will be used for other secondary endpoints. Cumulative incidence of healing at 12, 20 and 52 weeks post randomisation will be obtained from the primary endpoint analysis model. A Poisson-Gamma regression model will be fitted to infection status over time. A Cox Proportional Hazard's regression model will be fitted to time to re-ulceration of the index ulcer on those patients where healing of the index ulcer is confirmed. A repeated measures, random coefficients, linear regression model will be fitted to the DFS-SF score over time.

All adverse events and serious adverse events, including amputations and admissions to hospital, will be recorded and summarised by treatment strategy received. Expected treatment-related adverse events (AEs) include: pain, bleeding and infection from hydrosurgical debridement; bleeding, infection and skin irritation/breakdown from NPWT; seroma and allergic reaction from DCD.

Exploratory Analyses

Sensitivity analyses

For peer review only

For all analyses using the Cox Proportional Hazard's model, the assumption of independence of the distribution of time to healing /recurrence and time to other events, i.e. amputation and death will be assessed and alternative models considered if there are sufficient competing risks.

A multivariable Cox Proportional Hazards regression model will be fitted to explore risk factors predictive of time to healing.

Economic evaluation

A within-trial economic evaluation will be undertaken at week 52 post-randomisation. The proposed secondary endpoints and methods for the economic evaluation follow the reference case set out by NICE (34). The primary economic analysis will be a cost-utility analysis presenting incremental cost-effectiveness ratios (ICER) for each treatment strategy in rank order of effectiveness, with effects expressed in terms of quality-adjusted life years (QALY). An NHS and Personal Social Services (PSS) perspective for costs will be adopted. Costs and effects for each treatment strategy will be calculated for the trial follow-up period of 52 weeks.

Health resource use questionnaires will collect information on NHS and personal social care use in line with NICE guidelines (34). This will include primary, secondary, and community resource use. Unit cost data will be obtained from national databases such as the British National Formulary and Personal Social Services Research Unit (PSSRU) Costs of Health and Social Care.

Treatment costs include the cost of delivering each strategy (mainly given by person-time of health-care professionals) and the cost of the necessary equipment. The scope of resources considered includes the direct healthcare costs incurred for necessary patient care and excludes resources driven by the study protocol (e.g., routine clinics will be included, whilst research visits that are just for checking for re-ulceration are excluded; also, the cost of photography and visit time for collecting data for study purposes will be excluded). To cost the treatment strategies, data on average duration of appointments for delivering the treatment strategy will be collected.

Incremental cost-effectiveness ratios, Incremental Net Monetary Benefit (INMB) and Incremental Net Health Benefit (INHB) statistics will be computed. The National Institute for Health and Care Excellence (NICE) considers a cost per QALY within the range of £20,000-£30,000 to be acceptable (34).

Multiple Imputation will be used to address any issues of missing data in the base case analysis on the assumption of MAR. Complete Case analysis will be conducted as a sensitivity analysis.

Probabilistic Sensitivity Analysis (PSA) will be used to assess the impact of sampling uncertainty on the within trial evaluation results. Simulated cost and QALY estimates will be plotted on the cost-effectiveness plane to illustrate the uncertainty surrounding cost-effectiveness estimates (35) and presented as cost-effectiveness acceptability frontier (CEAF) to capture the varying probability of interventions being the most cost-effective over a range of willingness to pay for a QALY thresholds (36)

In addition, alternative scenarios will be explored in the sensitivity analysis to test the robustness of the main trial analysis results including analysis of complete cases only.

Data Management

For peer review only

Data will be monitored for quality and completeness and missing data will be chased up. Data received, including photographs will be stored in a secure database at Leeds CTRU in accordance with the 2018 Data Protection Act and the General Data Protection Regulation (GDPR).

Patient and Public Involvement

The trial was supported at the stage of developing the grant application by the Sheffield Teaching Hospitals Lay Advisory Panel for Diabetes & Endocrinology Research. Their input was central to study design, actively helping to shape discussions and decisions through written feedback and group discussion. In particular they informed decisions on the how frequently patients should be reassessed at the study site; whether patients would be willing to report healing or other outcomes directly to the research team or through their community clinician; agreement to completion of questionnaires, wound tracing and photographs which may be considered a burden; how willing patients will be to take part in the study; the acceptability of each intervention.

The trial has two PPI representatives on the Trial Steering Committee who have provided input into the patient information sheet and other trial documentation intended for use by patients. The PPI representatives also provide input into the design and conduct of the trial at 6 monthly meetings. This high-level involvement in project management aims to ensure patients perspectives are fully integrated in key decisions about the trial, delivery, and interpretation/dissemination of findings.

Ethics and Dissemination: Informed consent was provided by participants prior to commencement of their study. Ethics approval has been granted by NRES Committee Yorkshire & The Humber - Bradford Leeds Research Ethics Committee; approved 26th April 2017; (REC reference: 17/YH/0055). There is planned publication of a monograph in NIHR journals and main trial results and associated papers in high-impact peer reviewed journals.

Discussion

MAMS design:

The chosen MAMS design provides an efficient platform for assessment of several competing interventions, quickly homing in on the treatment strategy with greatest potential to be effective, early dropping of ineffective treatments and allowing assessment of combinations of treatments (20, 37-39). Specific advantages of the MAMS design include comparing multiple treatment arms to a shared control group thereby requiring fewer patients, improved consent/ recruitment rates since patients are more likely to receive an active treatment (39) and common eligibility criteria across trial arms (20). Moreover, recruitment to all treatment strategies will continue during analysis and reporting of Phase II data to ensure no loss of momentum in recruitment at sites.

Choice of endpoints:

Reduction in ulcer area at 4 weeks was chosen as the primary outcome at Phase II to be consistent with published observational studies (40, 41) and DFU RCTs (42). This intermediate outcome measure provides a means of screening for "emerging evidence" of efficacy, as it occurs earlier and more frequently than the definitive outcome measure and that it is on the causal pathway (43). Thus, it allows the Phase II analysis and decision on treatment selection to take place in a timely manner.

Time to healing, the primary endpoint in Phase III, was chosen as an important outcome measure from both clinical and economic perspectives (32).

Ulcer infection is the most common complication in non-healing ulcers, occurring in more than 50% of DFUs (6). It results in delayed healing, prolonged treatment, increased resource use and increases the risk of a patient requiring a major amputation (44). IDSA criteria is recognised as a gold standard for characterisation of infection in DFUs in many national and international guidelines and will provide a reproducible system for clinical diagnosis and severity stratification (12) (21) (45).

For peer review only

No single patient-reported outcome measure (PROM) has been identified as a “gold standard” for assessing HRQOL in diabetes-related foot disease (46). As a result, both the disease-specific questionnaire, DFS-SF (47) and the preference-based utility measure, EQ-5D-5L (www.euroqol.org) (48), will be completed by patients. The DFS-SF questionnaire has acceptable psychometric properties for measuring quality of life for patients with DFUs. The EQ-5D is a generic instrument and forms part of the NICE reference case for cost per Quality Adjusted Life Years (QALY) analysis.

Blinded assessment of healing

Having a blinded assessment of healing is important in reducing the risk of assessment bias and the trial includes independent, blinded clinical assessment of healing at both the first and confirmation of healing assessments, along with additional blinded review of photography undertaken by clinicians (CI (vascular surgeon) and clinical nurse specialists).

Revision to trial design

Since opening, the trial has undergone a trial re-design. The original design included a fourth treatment strategy in Phase II corresponding to a combination of HD and NPWT as an adjunct to TAU, and also allowed a maximum of two treatment strategies to go forward into Phase III under the same progression criteria. This original design required a maximum sample size of 660 participants, 324 participants recruited in Phase II and 336 in Phase III, under the sample size assumptions. Following a review of treatment strategies which would be considered in clinical practice if shown to be clinically and cost effective, a revised trial design dropped the combination of HD and NPWT arm, which reduced the maximum sample size to 447 patients whilst still ensuring a trial of clinical relevance.

Hard-to-heal ulcers

A registration phase was also included in the original trial design. It is important that the trial includes a patient population with “hard-to-heal” ulcers, reflecting the target population that would be considered for such adjuvant therapies in clinical practice. As there is variability in usual wound area assessment and documentation across recruiting centres, the registration phase included in the original design allowed a consistent approach to assessment of healing over a 4-week run-in period, and thereby considered “best practice” for trials of DFU healing. However, the recently published LeucoPatch trial (49), using an identical registration phase criterion of 50% healing at 4 weeks, reported only 22% healing in the control and 34% healing in the intervention arm, suggesting that this registration phase criteria is overly selective in the definition of “hard-to-heal”. Early audit of data from a single centre in MIDFUT showed that patients were not recruited to the trial at presentation to an MDT DFU clinic, and had been subjected to an “in house assessment period” prior to registration in the trial. 75% of those failing the trial registration period due to ulcer healing of >50% remained unhealed at 12 weeks, suggesting the trial was excluding a group of ulcers that were in fact “hard-to-heal”. The 2018 UK National Diabetic Foot Care Audit data of 21,000 ulcer episodes reports 49.3% remain unhealed at 12 weeks, with 27.3% unhealed 24 weeks and a further 2.9% recurring. Thus under half of ulcers unhealed at 12 weeks will heal in the subsequent 12 weeks, supporting a second inclusion criterion of ulcers that remain unhealed at a 12-week timepoint (9). A parallel entry route was therefore introduced to allow patients with ulcers of ≥12 weeks duration, that are considered hard-to-heal by the treating MDT team, to proceed directly to randomisation.

Following challenges in recruiting patients and the supporting evidence that a large proportion of patients with hard-to-heal ulcers were being missed, a decision was made to drop the Registration phase for all patients. Instead, included ulcers will have failed to reduce in area by >50% over at least 4 weeks as measured using local measurement techniques. This allows a more pragmatic approach to be taken in identifying patients with hard to heal ulcers by using local wound measurement policies, thereby minimises the risk of missing potentially eligible patients whilst ensuring the trial results are more generalisable to the target patient population.

For peer review only

Adjuvant therapies
It is not anticipated that there will be a rapid change in the technologies investigated in this trial, other than design changes aimed at increasing clinician and patient acceptance, again increasing the potential adoption and generalisability of the trial outcomes.

Trial Status
The first participant was registered on 10th August 2017 and the first participant randomised on 30th October 2017. As of 4th December 2019, 167 participants have been registered and 88 randomised. Recruitment is expected to complete by 31st August 2022. The full trial protocol is available on the NIHR journals library https://www.journalslibrary.nihr.ac.uk/programmes/hta/150877/#/.

Declarations

Data Availability
The datasets during and/or analysed during the current study will be available from the corresponding author on reasonable request.

Abbreviations

AE	Adverse Event
CEAF	Cost-Effectiveness Acceptability Frontier
CRF	Case Report Form
DCD	Decellularised Dermal Allograft
DMEC	Data Monitoring and Ethics Committee
DFU	Diabetic Foot Ulcer
DFS-SF	Diabetic Foot Scale-Short Form
eGFR	Estimated glomerular filtration rate
EQ-5D-5L	EuroQoL-5D 5 level
GDPR	General Data Protection Regulation
HbA1c	Haemoglobin A1c
HD	Hydrosurgical Debridement
HRQoL	Health Related Quality of Life
HRU	Health Resource Utilisation
HTA	Human Tissue Act
HTA	Health Technology Assessment
ICER	Incremental Cost Effective Ratio
IDSA	Infectious Diseases Society of America
INHB	Incremental Net Health Benefit
ITT	Intention to Treat
MAMS	Multi Arm Multi Stage
MAR	Missing At Random
MDT	Multidisciplinary team
NMB	Net Monetary Benefit
NHS	National Health Service
NHS BT	National Health Service Blood and Transplant
NICE	National Institute for Health and Care Excellence
NIHR	National Institute for Health Research
NPWT	Negative Pressure Wound Therapy
PROM	Patient Reported Outcome Measure
PSA	Probabilistic Sensitivity Analysis

For peer review only

PIL	Participant Information Leaflet
PSSRU	Personal Social Services Research Unit
QALY	Quality Adjusted Life Years
RCT	Randomised Controlled Trial
REC	Research Ethics Committee
RUSAE	Related Unexpected Serious Adverse Event
SAE	Serious Adverse Event
SWAT	Study Within a Trial
TAU	Treatment as Usual
TS	Treatment Strategy
TSC	Trial Steering Committee
WHO	World Health Organisation

Authors' contributions

DR, as the CI, conceived the trial, provided clinical expertise in the design and conduct of the trial, has overall leadership for the trial; JN provided expertise in the trial design and has scientific oversight; LS and JW provided senior statistical expertise in the trial design and have statistical and methodological oversight of the trial; SB provided statistical input on the trial design and is the supervising statistician, led on drafting the manuscript; MR is responsible for conducting the statistical analysis and reporting of the trial; RG is responsible for the coordination of the trial; CF is responsible for the overall management of the trial; HC is responsible for acquisition and management of the clinical data; ND and EM are Clinical Co-ordinators involving centre set-up support and ongoing monitoring; RL/CB has responsibility for the health economic design considerations and analysis; FG, EM, ND, PC, RL, SHV, IC, PV, EJ provided clinical expertise in the design and conduct of the trial; AC and RL provided expertise in the trial design and DCD. All authors reviewed draft versions and approved the final manuscript.

Funding Statement

This study was funded by the National Institute for Health Research (NIHR) Health Technology Assessment (HTA) Programme (Project: 15/08/77). The views and opinions expressed therein are those of the authors and do not necessarily reflect those of the HTA, NIHR, NHS or Department of Health.

We received product support for Versajet consoles and NPWT pumps from Smith & Nephew
We also received support for DCD from NHSBT and a £160K research support grant to cover excess treatment costs for consumables.

Competing interests

None: DR, FG, EJ, SHV, PC, JW, LS, RG, ND, EM, RLe, MR, HC, CF, JN, SB, CB, IC, RLon, PV

AC and RLom are employees of NHS BT who provide the DCD.

Acknowledgments

The authors would like to thank members of the Trial Steering Committee: Professor Simon Heller, Dr Jill Cundell, Professor Rob Hinchcliffe, Professor Steven Julious, Ms Brenda Riley, Ms Sonia Ward and in memory of Mr Paul Higgins; and members of the Data Monitoring and Ethics Committee: Mr Matt Sydes, Professor Julie Brittenden, Dr Magnus Löndahl, Mr Robert Davies and Professor Boby Mihaylova.

Licence Statement

I, the Submitting Author has the right to grant and does grant on behalf of all authors of the Work (as defined in the below author licence), an exclusive licence and/or a non-exclusive licence for contributions from authors who are: i) UK Crown employees; ii) where BMJ has agreed a CC-BY licence shall apply, and/or iii) in accordance

For peer review only

with the terms applicable for US Federal Government officers or employees acting as part of their official duties; on a worldwide, perpetual, irrevocable, royalty-free basis to BMJ Publishing Group Ltd ("BMJ") its licensees and where the relevant Journal is co-owned by BMJ to the co-owners of the Journal, to publish the Work in BMJ Open and any other BMJ products and to exploit all rights, as set out in our licence.

The Submitting Author accepts and understands that any supply made under these terms is made by BMJ to the Submitting Author unless you are acting as an employee on behalf of your employer or a postgraduate student of an affiliated institution which is paying any applicable article publishing charge ("APC") for Open Access articles. Where the Submitting Author wishes to make the Work available on an Open Access basis (and intends to pay the relevant APC), the terms of reuse of such Open Access shall be governed by a Creative Commons licence – details of these licences and which Creative Commons licence will apply to this Work are set out in our licence referred to above.

For peer review only

References:

1. IDF Diabetes Atlas. IDF Diabetes Altas eighth edition 2017. [Available from: https://www.idf.org/aboutdiabetes/what-is-diabetes/facts-figures.html.
2. IDF Diabetes Atlas. IDF Diabetes Altas sixth edition 2014 Update [02/12/2014]. 6:[Available from: http://www.idf.org/sites/default/files/Atlas-poster-2014_EN.pdf.
3. Abbott CA, Malik RA, van Ross ER, Kulkarni J, Boulton AJ. Prevalence and characteristics of painful diabetic neuropathy in a large community-based diabetic population in the UK. Diabetes care. 2011;34(10):2220-4.
4. Salvotelli L, Stoico V, Perrone F, Cacciatori V, Negri C, Brangani C, et al. Prevalence of neuropathy in type 2 diabetic patients and its association with other diabetes complications: The Verona Diabetic Foot Screening Program. Journal of diabetes and its complications. 2015;29(8):1066-70.
5. Singh N, Armstrong DG, Lipsky BA. Preventing foot ulcers in patients with diabetes. Jama-Journal of the American Medical Association. 2005;293(2):217-28.
6. Lavery LA, Armstrong DG, Wunderlich RP, Mohler MJ, Wendel CS, Lipsky BA. Risk factors for foot infections in individuals with diabetes. Diabetes Care. 2006;29(6):1288-93.
7. Armstrong DG, Lavery LA. Diabetic foot ulcers: Prevention, diagnosis and classification. American Family Physician. 1998;57(6):1325-32.
8. Diabetes UK https://www.diabetes.org.uk/Professionals/Position-statements-reports/Statistics/Diabetes-prevalence-2016/.
9. National Diabetes Foot Care Audit Report 2014-2017. https://files.digital.nhs.uk/pdf/e/9/ndfa-3ar-rep.pdf.
10. Kerr M (2017) Diabetic foot care in England: An economic study. https://www.diabetes.org.uk/Upload/Shared practice/Diabetic footcare in England, An economic case study (January 2017).pdf.
11. Hogg F, Peach G, Price P, Thompson M, Hinchliffe R. Measures of health-related quality of life in diabetes-related foot disease: a systematic review. Diabetologia. 2012;55(3):552-65.
12. NICE NG19. Diabetic foot problems: prevention and management 2015. Available from: https://www.nice.org.uk/guidance/ng19.
13. National Diabetes Foot Care Audit Report 2015-2018 https://files.digital.nhs.uk/50/8E75BA/NDFA%204AR%20-%20Main%20Report%20v1.0.pdf accessed 5/11/19.
14. Sheehan P, Jones P, Caselli A, Giurini JM, Veves A. Percent change in wound area of diabetic foot ulcers over a 4-week period is a robust predictor of complete healing in a 12-week prospective trial. Diabetes Care. 2003;26(6):1879-82.
15. Guest JF, Fuller GW, Vowden P. Diabetic foot ulcer management in clinical practice in the UK: costs and outcomes. International wound journal. 2018;15(1):43-52.
16. Dumville JC, Hinchliffe RJ, Cullum N, Game F, Stubbs N, Sweeting M, et al. Negative pressure wound therapy for treating foot wounds in people with diabetes mellitus. Cochrane Database of Systematic Reviews. 2013(10).
17. Caputo WJ, Beggs DJ, DeFede JL, Simm L, Dharma H. A prospective randomised controlled clinical trial comparing hydrosurgery debridement with conventional surgical debridement in lower extremity ulcers. International Wound Journal. 2008;5(2):288-94.
18. Brigido SA. The use of an acellular dermal regenerative tissue matrix in the treatment of lower extremity wounds: a prospective 16-week pilot study. International Wound Journal. 2006;3(3):181-7.
19. Reyzelman A, Crews RT, Moore JC, Moore L, Mukker JS, Offutt S, et al. Clinical effectiveness of an acellular dermal regenerative tissue matrix compared to standard wound management in healing diabetic foot ulcers: a prospective, randomised, multicentre study. International Wound Journal. 2009;6(3):196-208.

For peer review only

20. Pallmann P, Bedding AW, Choodari-Oskooei B, Dimairo M, Flight L, Hampson LV, et al. Adaptive designs in clinical trials: why use them, and how to run and report them. BMC Medicine. 2018;16(1):29.
21. Game FL AJ, Attinger C, Hartemann A, Hinchliffe RJ, Löndahl M, Price PE, Jeffcoate WJ on behalf of the International Working Group on the Diabetic Foot (IWGDF). . Effectiveness of interventions to enhance healing of chronic ulcers of the foot in diabetes: a systematic review. Diabetes/Metabolism Research and Reviews. 2016;32(S1):154-68.
22. World Health Organisation. Definition and Diagnosis of Diabetes Mellitus and Intermediate Hyperglycemia 2006 [06/10/2016]. Available from: http://apps.who.int/iris/bitstream/10665/43588/1/9241594934_eng.pdf.
23. Lipsky BA, Berendt AR, Cornia PB, Pile JC, Peters EJG, Armstrong DG, et al. 2012 Infectious Diseases Society of America Clinical Practice Guideline for the Diagnosis and Treatment of Diabetic Foot Infections. Clinical Infectious Diseases. 2012;54(12):E132-U232.
24. Dunnett CW. A multiple comparison procedure for comparing several treatments with a control. Journal of the American Statistical Association. 1955;50(272):1096-121.
25. Schneider CA, Rasband WS, Eliceiri KW. NIH Image to ImageJ: 25 years of image analysis. Nature methods. 2012;9(7):671-5.
26. Ince P, Abbas ZG, Lutale JK, Basit A, Ali SM, Chohan F, et al. Use of the SINBAD classification system and score in comparing outcome of foot ulcer management on three continents. Diabetes care. 2008;31(5):964-7.
27. J Jeffcoate W, Bus S, Game F, J Hinchliffe R, Price P, Schaper N. Reporting standards of studies and papers on the prevention and management of foot ulcers in diabetes: required details and markers of good quality2016.
28. Peters EJ, Armstrong DG, Lavery LA. Risk factors for recurrent diabetic foot ulcers: site matters. Diabetes Care. 2007;30(8):2077-9.
29. Edmonds M, European Australian A. Apligraf in the Treatment of Neuropathic Diabetic Foot Ulcers. International Journal of Lower Extremity Wounds. 2009;8(1):11-8.
30. Veves A, Falanga V, Armstrong DG, Sabolinski ML, Apligraf Diabetic Foot Ulcer S. Graftskin, a human skin equivalent, is effective in the management of noninfected neuropathic diabetic foot ulcers - A prospective randomized multicenter clinical trial. Diabetes Care. 2001;24(2):290-5.
31. Veves A, Sheehan P, Pham HT, Promogran Diabetic Foot Ulcer S. A randomized, controlled trial of promogran (a collagen/oxidized regenerated cellulose dressing) vs standard treatment in the management of diabetic foot ulcers. Archives of Surgery. 2002;137(7):822-7.
32. Gottrup F, Cullen BM, Karlsmark T, Bischoff-Mikkelsen M, Nisbet L, Gibson MC. Randomized controlled trial on collagen/oxidized regenerated cellulose/silver treatment. Wound Repair and Regeneration. 2013;21(2):216-25.
33. White IR, Carpenter J, Horton NJ. Including all individuals is not enough: lessons for intention-to-treat analysis. Clinical trials. 2012;9(4):396-407.
34. NICE. Guide to the methods of technology appraisal 2013 [09/10/2016]. Available from: https://www.nice.org.uk/process/pmg9/chapter/foreword#discounting-2.
35. O'Brien BJ, Briggs AH. Analysis of uncertainty in health care cost-effectiveness studies: an introduction to statistical issues and methods. Statistical Methods in Medical Research. 2002;11(6):455-68.
36. Fenwick E, O'Brien BJ, Briggs A. Cost-effectiveness acceptability curves - facts, fallacies and frequently asked questions. Health Economics. 2004;13(5):405-15.
37. Wason JMS, Jaki T. Optimal design of multi-arm multi-stage trials. Statistics in Medicine. 2012;31(30):4269-79.
38. Schmidli H, Bretz F, Racine A, Maurer W. Confirmatory Seamless Phase II/III Clinical Trials with Hypotheses Selection at Interim: Applications and Practical Considerations. Biometrical Journal. 2006;48(4):635-43.

For peer review only

39. Parmar MKB, Carpenter J, Sydes MR. More multiarm randomised trials of superiority are needed. The Lancet. 2014;384(9940):283-4.
40. Margolis DJ, Gelfand JM, Hoffstad O, Berlin JA. Surrogate end points for the treatment of diabetic neuropathic foot ulcers. Diabetes care. 2003;26(6):1696-700.
41. Snyder RJ, Cardinal M, Dauphinee DM, Stavosky J. A Post-hoc Analysis of Reduction in Diabetic Foot Ulcer Size at 4 Weeks as a Predictor of Healing by 12 Weeks. Ostomy Wound Management. 2010;56(3):44-+.
42. Game F, Jeffcoate W, Tarnow L, Day F, Fitzsimmons D, Jacobsen J. The LeucoPatch® system in the management of hard-to-heal diabetic foot ulcers: study protocol for a randomised controlled trial. Trials. 2017;18(1):469.
43. Sydes MR, Parmar MK, James ND, Clarke NW, Dearnaley DP, Mason MD, et al. Issues in applying multi-arm multi-stage methodology to a clinical trial in prostate cancer: the MRC STAMPEDE trial. Trials. 2009;10(1):39.
44. Pickwell K, Siersma V, Kars M, Apelqvist J, Bakker K, Edmonds M, et al. Predictors of lower-extremity amputation in patients with an infected diabetic foot ulcer. Diabetes Care. 2015;38(5):852-7.
45. Lipsky BA, Aragón-Sánchez J, Diggle M, Embil J, Kono S, Lavery L, et al. IWGDF guidance on the diagnosis and management of foot infections in persons with diabetes. Diabetes/metabolism research and reviews. 2016;32:45-74.
46. Hogg FRA, Peach G, Price P, Thompson MM, Hinchliffe RJ. Measures of health-related quality of life in diabetes-related foot disease: a systematic review. Diabetologia. 2012;55(3):552-65.
47. Bann CM, Fehnel SE, Gagnon DD. Development and validation of the Diabetic Foot Ulcer Scale-short form (DFS-SF). Pharmacoeconomics. 2003;21(17):1277-90.
48. EuroQol Group. What is EQ-5D 2015 [09/10/2015]. Available from: www.euroqol.org/eq-5d/what-is-eq-5d.
49. Game F, Jeffcoate W, Tarnow L, Jacobsen JL, Whitham DJ, Harrison EF, et al. LeucoPatch system for the management of hard-to-heal diabetic foot ulcers in the UK, Denmark, and Sweden: an observer-masked, randomised controlled trial. The lancet Diabetes & endocrinology. 2018;6(11):870-8.

Figure Legends:

Figure 1: MIDFUT trial design

Figure 2: Flow Diagram

For peer review only

Figure 1: MIDFUT trial design

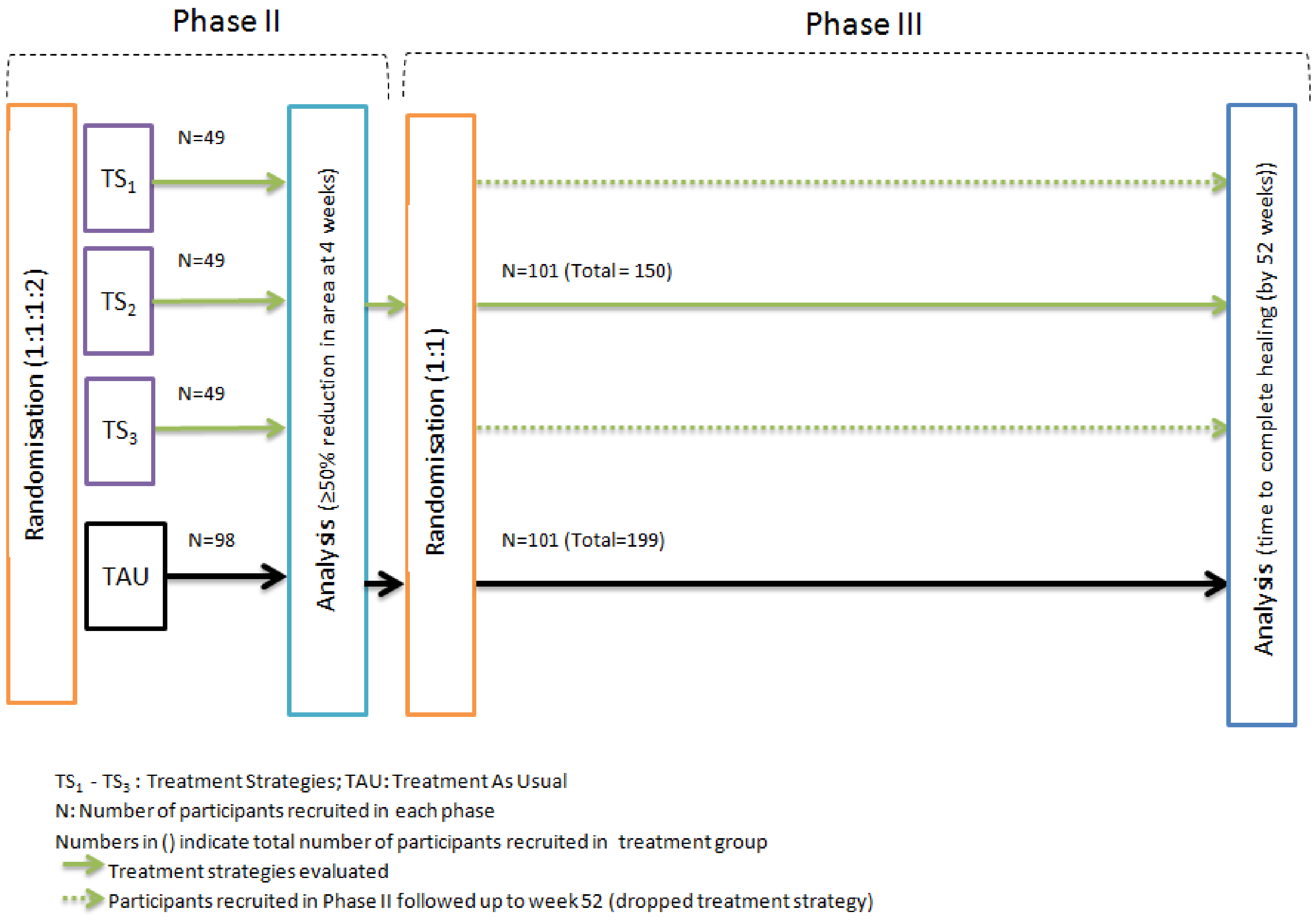

Figure 2: Flow Diagram

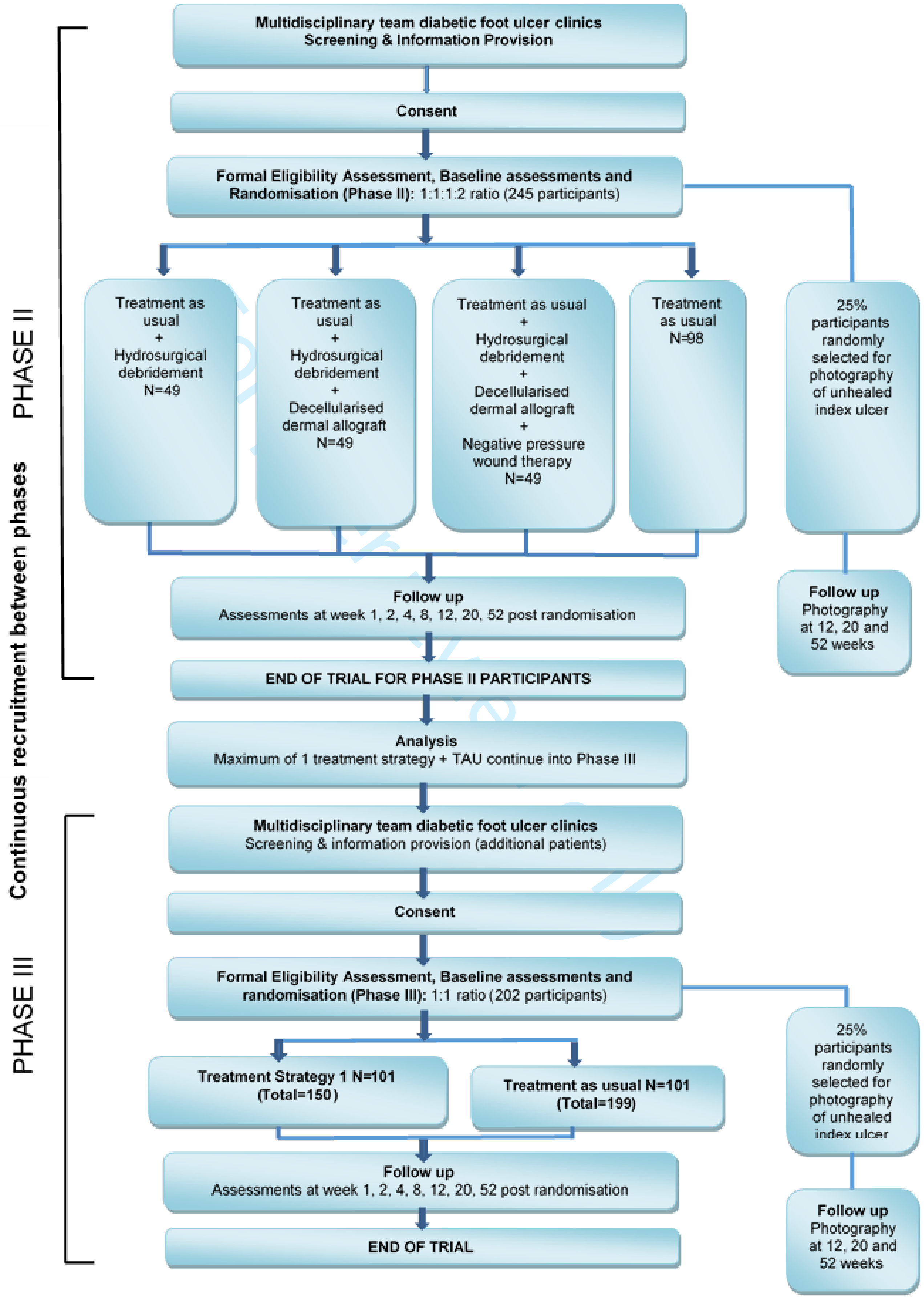

STANDARD PROTOCOL ITEMS: RECOMMENDATIONS FOR INTERVENTIONAL TRIALS

SPIRIT 2013 Checklist: Recommended items to address in a clinical trial protocol and related documents*

Section/item	Item No	Description	
**Administrative information**			
Title	1	Descriptive title identifying the study design, population, interventions, and, if applicable, trial acronym	Page 1
Trial registration	2a	Trial identifier and registry name. If not yet registered, name of intended registry	Page 2
	2b	All items from the World Health Organization Trial Registration Data Set	N/A
Protocol version	3	Date and version identifier	Page 16
Funding	4	Sources and types of financial, material, and other support	Page 17
Roles and responsibilities	5a	Names, affiliations, and roles of protocol contributors	Pages 1, 17
	5b	Name and contact information for the trial sponsor	Page 16 (see link to Protocol)
	5c	Role of study sponsor and funders, if any, in study design; collection, management, analysis, and interpretation of data; writing of the report; and the decision to submit the report for publication, including whether they will have ultimate authority over any of these activities	Page 17
	5d	Composition, roles, and responsibilities of the coordinating centre, steering committee, endpoint adjudication committee, data management team, and other individuals or groups overseeing the trial, if applicable (see Item 21a for data monitoring committee)	Page 17
**Introduction**			
Background and rationale	6a	Description of research question and justification for undertaking the trial, including summary of relevant studies (published and unpublished) examining benefits and harms for each intervention	Pages 3 & 4
	6b	Explanation for choice of comparators	Pages 3 &4

For peer review only

Objectives	7	Specific objectives or hypotheses	Pages 4 & 5
Trial design	8	Description of trial design including type of trial (eg, parallel group, crossover, factorial, single group), allocation ratio, and framework (eg, superiority, equivalence, noninferiority, exploratory)	Pages 5 & 7

Methods: Participants, interventions, and outcomes

Study setting	9	Description of study settings (eg, community clinic, academic hospital) and list of countries where data will be collected. Reference to where list of study sites can be obtained	Page 6
Eligibility criteria	10	Inclusion and exclusion criteria for participants. If applicable, eligibility criteria for study centres and individuals who will perform the interventions (eg, surgeons, psychotherapists)	Pages 6-8
Interventions	11a	Interventions for each group with sufficient detail to allow replication, including how and when they will be administered	Pages 8&9
	11b	Criteria for discontinuing or modifying allocated interventions for a given trial participant (eg, drug dose change in response to harms, participant request, or improving/worsening disease)	N/A
	11c	Strategies to improve adherence to intervention protocols, and any procedures for monitoring adherence (eg, drug tablet return, laboratory tests)	N/A
	11d	Relevant concomitant care and interventions that are permitted or prohibited during the trial	N/A
Outcomes	12	Primary, secondary, and other outcomes, including the specific measurement variable (eg, systolic blood pressure), analysis metric (eg, change from baseline, final value, time to event), method of aggregation (eg, median, proportion), and time point for each outcome. Explanation of the clinical relevance of chosen efficacy and harm outcomes is strongly recommended	Pages 4, 5 & 8
Participant timeline	13	Time schedule of enrolment, interventions (including any run-ins and washouts), assessments, and visits for participants. A schematic diagram is highly recommended (see Figure)	Pages 8-10
Sample size	14	Estimated number of participants needed to achieve study objectives and how it was determined, including clinical and statistical assumptions supporting any sample size calculations	Page 10
Recruitment	15	Strategies for achieving adequate participant enrolment to reach target sample size	Pages 6 & 7

Methods: Assignment of interventions (for controlled trials)

Allocation:

For peer review only - http://bmjopen.bmj.com/site/about/guidelines.xhtml

Sequence generation	16a	Method of generating the allocation sequence (eg, computer-generated random numbers), and list of any factors for stratification. To reduce predictability of a random sequence, details of any planned restriction (eg, blocking) should be provided in a separate document that is unavailable to those who enrol participants or assign interventions	Page 7
Allocation concealment mechanism	16b	Mechanism of implementing the allocation sequence (eg, central telephone; sequentially numbered, opaque, sealed envelopes), describing any steps to conceal the sequence until interventions are assigned	Page 7
Implementation	16c	Who will generate the allocation sequence, who will enrol participants, and who will assign participants to interventions	Page 7
Blinding (masking)	17a	Who will be blinded after assignment to interventions (eg, trial participants, care providers, outcome assessors, data analysts), and how	Page 8
	17b	If blinded, circumstances under which unblinding is permissible, and procedure for revealing a participant's allocated intervention during the trial	N/A
**Methods: Data collection, management, and analysis**			
Data collection methods	18a	Plans for assessment and collection of outcome, baseline, and other trial data, including any related processes to promote data quality (eg, duplicate measurements, training of assessors) and a description of study instruments (eg, questionnaires, laboratory tests) along with their reliability and validity, if known. Reference to where data collection forms can be found, if not in the protocol	Pages 9, 10 & 14
	18b	Plans to promote participant retention and complete follow-up, including list of any outcome data to be collected for participants who discontinue or deviate from intervention protocols	N/A
Data management	19	Plans for data entry, coding, security, and storage, including any related processes to promote data quality (eg, double data entry; range checks for data values). Reference to where details of data management procedures can be found, if not in the protocol	Page 14
Statistical methods	20a	Statistical methods for analysing primary and secondary outcomes. Reference to where other details of the statistical analysis plan can be found, if not in the protocol	Pages 12 & 13
	20b	Methods for any additional analyses (eg, subgroup and adjusted analyses)	Page 13
	20c	Definition of analysis population relating to protocol non-adherence (eg, as randomised analysis), and any statistical methods to handle missing data (eg, multiple imputation)	Page 12

For peer review only - http://bmjopen.bmj.com/site/about/guidelines.xhtml

Methods: Monitoring

Data monitoring	21a	Composition of data monitoring committee (DMC); summary of its role and reporting structure; statement of whether it is independent from the sponsor and competing interests; and reference to where further details about its charter can be found, if not in the protocol. Alternatively, an explanation of why a DMC is not needed	Page 11
	21b	Description of any interim analyses and stopping guidelines, including who will have access to these interim results and make the final decision to terminate the trial	Page 11
Harms	22	Plans for collecting, assessing, reporting, and managing solicited and spontaneously reported adverse events and other unintended effects of trial interventions or trial conduct	Page 12
Auditing	23	Frequency and procedures for auditing trial conduct, if any, and whether the process will be independent from investigators and the sponsor	N/A

Ethics and dissemination

Research ethics approval	24	Plans for seeking research ethics committee/institutional review board (REC/IRB) approval	Pages 2 & 14
Protocol amendments	25	Plans for communicating important protocol modifications (eg, changes to eligibility criteria, outcomes, analyses) to relevant parties (eg, investigators, REC/IRBs, trial participants, trial registries, journals, regulators)	N/A
Consent or assent	26a	Who will obtain informed consent or assent from potential trial participants or authorised surrogates, and how (see Item 32)	Pages 6 & 7
	26b	Additional consent provisions for collection and use of participant data and biological specimens in ancillary studies, if applicable	N/A
Confidentiality	27	How personal information about potential and enrolled participants will be collected, shared, and maintained in order to protect confidentiality before, during, and after the trial	Page 14
Declaration of interests	28	Financial and other competing interests for principal investigators for the overall trial and each study site	Page 17
Access to data	29	Statement of who will have access to the final trial dataset, and disclosure of contractual agreements that limit such access for investigators	Page 16
Ancillary and post-trial care	30	Provisions, if any, for ancillary and post-trial care, and for compensation to those who suffer harm from trial participation	N/A

For peer review only

Dissemination policy	31a	Plans for investigators and sponsor to communicate trial results to participants, healthcare professionals, the public, and other relevant groups (eg, via publication, reporting in results databases, or other data sharing arrangements), including any publication restrictions	Page 14
	31b	Authorship eligibility guidelines and any intended use of professional writers	N/A
	31c	Plans, if any, for granting public access to the full protocol, participant-level dataset, and statistical code	Page 16
**Appendices**			
Informed consent materials	32	Model consent form and other related documentation given to participants and authorised surrogates	N/A
Biological specimens	33	Plans for collection, laboratory evaluation, and storage of biological specimens for genetic or molecular analysis in the current trial and for future use in ancillary studies, if applicable	N/A

*It is strongly recommended that this checklist be read in conjunction with the SPIRIT 2013 Explanation & Elaboration for important clarification on the items. Amendments to the protocol should be tracked and dated. The SPIRIT checklist is copyrighted by the SPIRIT Group under the Creative Commons "Attribution-NonCommercial-NoDerivs 3.0 Unported" license.

For peer review only
