# Supplementary material for: Multiple Interventions for Diabetic Foot Ulcer Treatment Trial (MIDFUT): study protocol for a randomised controlled trial
Source: BMJ Open. 2020 Apr 19;10(4):e035947. doi: 10.1136/bmjopen-2019-035947 (PMC7245399; doi:10.1136/bmjopen-2019-035947)
Supplement: Reviewer comments [file bmjopen-2019-035947.reviewer_comments.pdf]

PEER REVIEW HISTORY

BMJ Open publishes all reviews undertaken for accepted manuscripts. Reviewers are asked to complete a checklist review form (**http://bmjopen.bmj.com/site/about/resources/checklist.pdf**) and are provided with free text boxes to elaborate on their assessment. These free text comments are reproduced below.

ARTICLE DETAILS

**TITLE (PROVISIONAL)**	Multiple Interventions for Diabetic Foot Ulcer Treatment Trial (MIDFUT): Study Protocol for a randomised controlled trial
**AUTHORS**	Brown, Sarah; Nixon, Jane; Ransom, Myka; Gilberts, Rachael; Dewhirst, Nikki; McGinnis, Elizabeth; Longo, Roberto; Game, Frances; Bojke, Chris; Chadwick, Paul; Chandrasekar, Akila; Chetter, IC; Collier, Howard; Fernandez, Catherine; Homer-Vanniasinkam, Shervanthi; Jude, Edward; Leigh, Richard; Lomas, Richard; Vowden, Peter; Wason, James; Sharples, Linda; Russell, David

This article was not externally reviewed at BMJ Open. Protocols that have been independently assessed prior to submission to BMJ Open are usually fast-tracked to publication on the grounds that further substantial changes will not be possible. This independent assessment will usually be external, independent review for both a substantial grant award from a non-commercial or government funder and ethics approval.
